# Role of mouse adenovirus type 1 E4orf6-induced degradation of protein kinase R in pathogenesis

**DOI:** 10.1128/jvi.01545-24

**Published:** 2024-12-31

**Authors:** Daniel F. Edwards, III, Estela A. Pereira, Luiza A. Castro-Jorge, Juan M. Nevarez, Oded Foreman, Katherine R. Spindler

**Affiliations:** 1 Department of Microbiology and Immunology, University of Michigan 242912 , Ann Arbor, Michigan, USA; 2 Department of Pathology, Genentech, Inc. 7412 , South San Francisco, California, USA; International Centre for Genetic Engineering and Biotechnology, Trieste, Italy

**Keywords:** protein kinase R, PKR, viral pathogenesis, proteasome, Cullin

## Abstract

Protein kinase R (PKR) is an interferon-induced antiviral protein activated by autophosphorylation in response to double strand DNA (dsRNA) and other stimuli. Activated PKR causes translation inhibition and apoptosis, and it contributes to proinflammatory responses, cell growth, and differentiation. Mouse adenovirus type 1 (MAV-1) counteracts PKR by causing its degradation via a viral protein, early region 4 open reading frame 6 (E4orf6). Degradation is dependent on E4orf6 binding to Cullin 2, a component of the MAV-1 E4orf6 ubiquitin ligase. We investigated the importance of E4orf6 for induction of PKR degradation by exploiting the ability to infect the natural host with the adenovirus MAV-1. First, we used a new PKR-deficient mouse strain, PKR-TKO. PKR-TKO mouse embryo fibroblasts (MEFs) produced higher levels of MAV-1 upon infection than did wild-type (WT) MEFs. PKR-TKO mice had significantly reduced survival, and MAV-1 had a lower LD_50_ than in WT control mice. However, virus loads in brains and spleens, key organs infected by MAV-1, were similar between PKR-TKO and WT mice. Second, we constructed a virus, E4orf6TMC2, that has three amino acid changes in the E4orf6 domain involved in Cullin 2 binding. In cell culture infection, compared to WT virus, E4orf6TMC2 resulted in reduced PKR degradation, but its growth was equivalent to WT virus. However, E4orf6TMC2 was avirulent in three mouse strains, including the PKR-TKO mice. The results indicate that PKR is an essential antiviral protein that protects against MAV-1 infection. We confirmed that the viral E4orf6 protein is a virulence protein important for PKR degradation during virus infection, and our results suggest its function is not limited to PKR degradation.

**IMPORTANCE:**

Protein kinase R (PKR) is a host protein that is central to many aspects of the cellular stress response. PKR protects against viral infection by inhibiting viral and host protein synthesis. Most animal viruses have developed ways to circumvent PKR effects by at least one of a variety of means, including inducing its degradation. A new mouse strain knocked out for PKR expression has enabled us to show the importance of PKR for protection from mouse adenovirus type 1 infection in the natural host, which is not possible for human adenoviruses. Mouse adenovirus type 1 induces degradation of PKR through an interaction with host protein Cullin 2. We generated a mutant virus that is defective in its ability to interact with Cullin 2 and showed that the virus does not cause pathogenesis in mice. This work provides critical evidence from mouse studies supporting the importance of PKR for adenovirus pathogenesis.

## INTRODUCTION

A major host response to viral infection is the activation of protein kinase R (PKR), an interferon-stimulated gene product that phosphorylates the translation initiation factor eukaryotic initiation factor 2 α (eIF2α) in response to a dsRNA stimulus, leading to protein synthesis arrest and formation of stress granules (reviewed in references [1–3]). In addition to this well-known antiviral activity limiting translation and consequently viral replication, PKR has many regulatory functions in cellular processes that are likely to affect the course of viral infection, including activation of apoptosis, signal transduction of the inflammatory response, innate immune signaling, and growth regulation and differentiation (reviewed in references [1–4]). It is thus not surprising that RNA and DNA viruses have a wide range of mechanisms to overcome PKR activation and eIF2α phosphorylation, including producing viral factors that inhibit by directly binding PKR, causing PKR degradation, altering its subcellular localization, inhibiting its kinase function, and/or altering its interaction with eIF2α. Human adenoviruses (HAdVs) produce small single strand RNA (ssRNA) transcripts that have extensive intramolecular base pairing, called virus-associated (VA) RNAs, which bind PKR and block its activation by dsRNA (5). In contrast, mouse adenovirus type 1 (MAV-1, also known as murine adenovirus, MAdV-1) does not produce VA-like RNAs but causes PKR degradation through a proteasomal mechanism involving the viral early region 4 open reading frame 6 (E4orf6) protein (6, 7).

MAV-1 affords study of adenovirus pathogenesis in a tractable small animal model. MAV-1 has similarities to HAdVs in overall organization of its ~31 kb dsDNA genome, transcription map, and the functions of some early genes (8, 9). This has enabled the characterization of adenovirus molecular biology and pathogenesis in cell culture and mice and its development as a vector for exogenous genes (10). There are some notable differences between MAV-1 and HAdVs, including differences in countering the host antiviral PKR response, as noted above. In addition, there are different numbers and organization of early genes encoded by early regions 1, 3, and 4 (E1, E3, and E4), while the E2 region, which encodes the DNA replication machinery, is conserved (11–13). HAdVs typically infect epithelial cells of the respiratory tract, gastrointestinal tract, and the eye (reviewed in reference [14]), whereas MAV-1 infects endothelial cells and monocytes/macrophages throughout the animal, myocytes, and astrocytes in the central nervous system (CNS; reviewed in reference [9]). MAV-1 can infect epithelial cells when inoculated intranasally (15, 16). Consistent with their tropism, HAdVs cause respiratory illness, conjunctivitis, gastroenteritis, and, less often, pneumonia and myocarditis in immunocompetent individuals (17). In contrast, MAV-1 causes pantropic infections, and high levels of virus are found in the CNS and spleen, characterized by encephalitis and disruption of the blood-brain barrier, and MAV-1 also causes respiratory infections and myocarditis (9). Thus, it is not surprising that the mechanisms by which some genes interact with the host may differ between MAV-1 and HAdVs.

The E4 regions of HAdVs produce a variety of spliced mRNAs, which in the case of HAdV-5, are predicted to encode at least seven proteins (reviewed in reference [17, 18]). HAdV-5 E4orf6 and E1B 55K are well-established protein partners that lead to polyubiquitination of cellular proteins and their subsequent degradation (17). These degraded proteins include p53, proteins in a complex involved in sensing DNA damage, and additional proteins involved in the DNA damage response that is induced by adenovirus infection. MAV-1 E4 also has variant spliced mRNAs; although long-read direct DNA sequencing has not been performed, sequencing of cDNAs identified six spliced forms with the potential to encode at least three proteins (13), with sequence similarity to HAdV-2 E4orf6, orf3, and orf6/7 (13, 19). The predicted MAV-1 E4orf6 protein has 48% amino acid similarity to the HAdV-2 orf6-encoded 34K protein (19). HAdV-2 and MAV-1 E4orf6 also have functional similarity. In human cells, MAV-1 E4orf6 coimmunoprecipitates with host cell proteins Cullin 2 (Cul2) and Elongin C to form a ubiquitin ligase complex, and MAV-1 E1B 55K and E4orf6 together result in degradation of mouse p53 (20). Also in human cells, MAV-1 E4orf6 binds human DNA ligase IV, involved in the DNA damage response, causing its degradation in the absence of MAV-1 E1B 55K protein. Degradation of mouse PKR in mouse cells in culture requires only the MAV-1 E4orf6 protein and not the E1B 55K protein (7). In mouse cells infected by MAV-1, p53 is ubiquitinated, but we were unable to detect ubiquitination of PKR (6). However, degradation of PKR *in vitro* requires the MAV-1 E4orf6 domain involved in binding Cul2 and is inhibited by proteasome inhibitors MG132 and bortezomib, consistent with PKR degradation being carried out by a proteasomal mechanism (6, 7).

Mice with defects in PKR expression and cells from those mice have been used to investigate pathogenesis of many viruses. Two commonly used mouse strains thought to be PKR knockouts either have an N-terminal deletion of PKR or a C-terminal deletion in the PKR protein (21, 22). In some cell culture conditions, cells from both strains of mice can express a truncated form of PKR, which may complicate interpretations of their use (23). To circumvent this, we produced PKR knockout mice from sperm that were CRISPR/Cas9-edited in the eIF2ak2 (PKR) gene, and we designated them PKR-TKO mice (7). While the earlier PKR knockout mice expressed truncated proteins, PKR expression is undetectable in our PKR-TKO mice.

In this report, we have characterized the interaction of MAV-1 and PKR using PKR-TKO mouse embryo fibroblasts (MEFs) and PKR-TKO mice. Yields of infectious virus were higher in PKR-TKO MEFs than wild-type (WT) MEFs. PKR-TKO mice infected intraperitoneally (i.p.) with WT MAV-1 had significantly reduced survival compared to WT mice. These data indicate that PKR is an antiviral factor in MAV-1 infections. No difference was seen between WT and PKR-TKO mice in virus loads in infected brains or spleens. Although expression of chemokines CCL2 and CXCL10 was increased upon MAV-1 infection, there were no differences between WT and PKR-TKO mice in these chemokine levels. We also did not see a difference between WT and PKR-TKO mice in permeability to sodium fluorescein, a proxy for blood-brain barrier disruption. To examine the interaction of MAV-1 and PKR further, we constructed a virus with amino acid changes in the domain of E4orf6 that interacts with Cul2, which is important for the proteasomal degradation of PKR (7). This mutant virus produced virus yields in mouse 3T6 cells equivalent to WT virus. However, mutant virus infection of 3T6 cells resulted in less PKR degradation than WT virus infection, as expected based on the amino acid changes and previous transfection results (7). Infections of three strains of inbred mice, including one lacking PKR, indicated that the E4orf6 mutant virus was less virulent than WT virus *in vivo*; no mice died at the highest dose possible. We report the results of other assays comparing mice infected with WT and the E4orf6 mutant virus. Taken together, the results show that PKR is a protective antiviral factor in MAV-1 infection, and functional MAV-1 E4orf6 is important for PKR degradation *in vitro* and for virulence in mice. The results further suggest that MAV-1 E4orf6 has important functions in addition to causing degradation of PKR.

## RESULTS

### Absence of PKR results in increased MAV-1 yield in cultured MEFs

**Fig 1:**
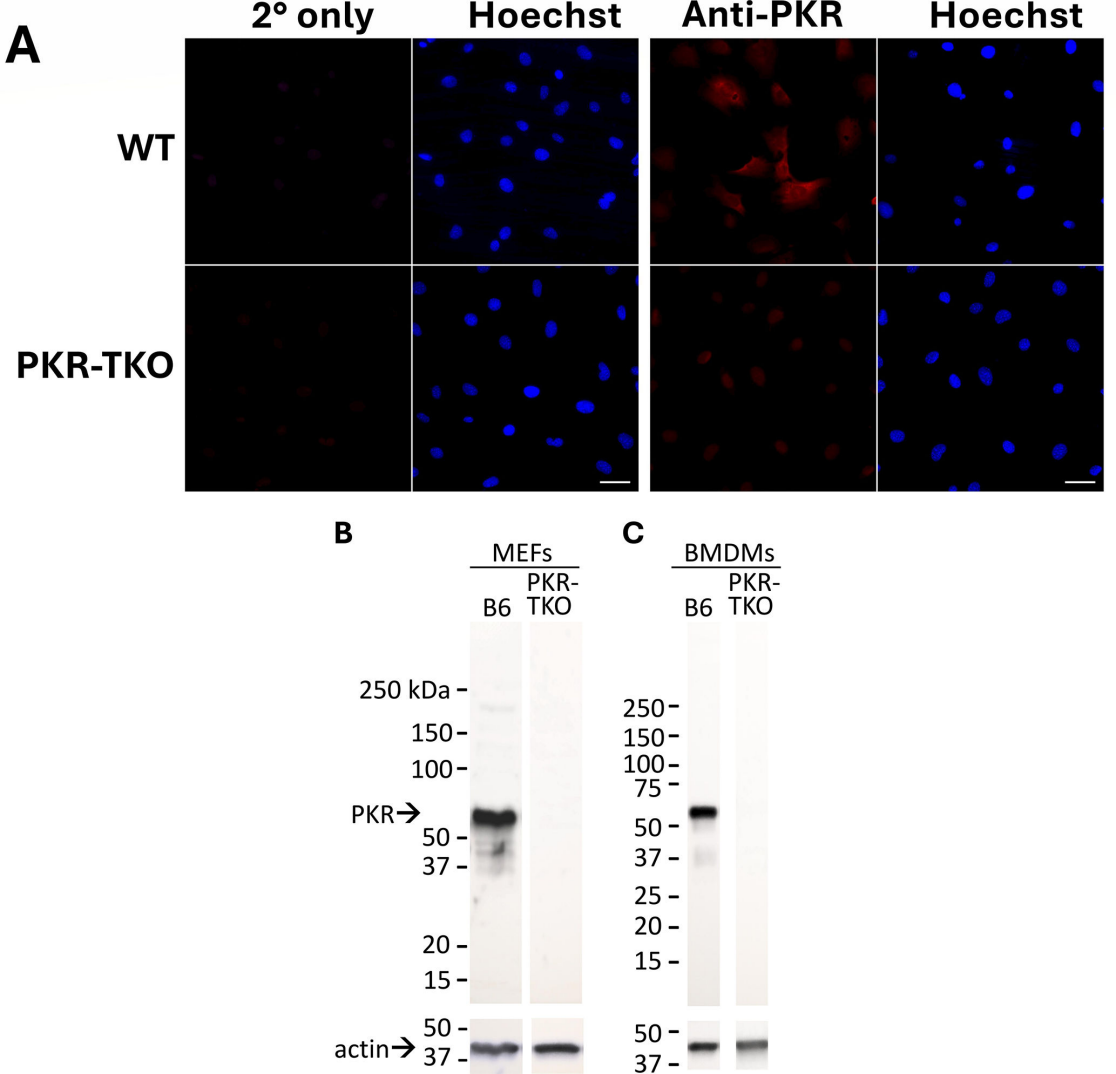
PKR-TKO MEFs and BMDMs do not express PKR. Cells were plated in 24-well plates (**A**) at 3.5 × 10^4^ cells/well or in 12-well plates at 3 × 10^5^ cells/well (MEFs, **B**), or 1 × 10^6^ cells/well (BMDMs, **C**). (**A**) Immunofluorescence staining. Cells were fixed, permeabilized, blocked, and stained for PKR (red) and DNA (Hoechst, blue). Samples were probed with rabbit anti-PKR EPR19374 (Abcam) at 1:50 and secondary anti-rabbit Alexa Fluor 647 at 1:500. Secondary-only controls (2° only) omitted the anti-PKR antibody. Magnification: 40×. Scale bar: 50 µM. (**B and C**) Immunoblot analysis. Cells (B6 or PKR-TKO MEFs or BMDMs) were plated in 12-well plates. Total lysates were analyzed by immunoblot using anti-PKR B-10 at 1:200 and secondary antibody at 1:1,000 (top). Anti-β-actin antibody at 1:1,000 was used as a loading control (bottom). Molecular weight markers and positions of the PKR and actin bands are indicated on the left.

We previously generated a mouse strain knocked out for PKR and then generated fibroblasts from day 15 embryos (PKR-TKO MEFs) (7). We also prepared WT day 15 MEFs (WT MEFs) from a C57BL/6 control mouse bred in the same animal room. We demonstrated that the PKR-TKO MEFs do not make PKR that is detectable by immunofluorescence or immunoblot, as follows. WT and PKR-TKO MEFs were plated on coverslips or 100 mm plates. After 24 h, coverslips were analyzed by immunofluorescence as described in Materials and Methods using a rabbit anti-PKR monoclonal antibody. The results showed that PKR-TKO MEFs did not express detectable PKR, whereas WT MEFs did express PKR (Fig. 1A). Negative controls were performed by incubating cells with secondary antibody only, under equivalent assay conditions. The cells showed no immunofluorescence. For immunoblot analysis, extracts were prepared from WT or PKR-TKO MEFs and a second cell type, bone marrow-derived macrophages (BMDMs), cultured in 12-well plates. We analyzed the extracts by immunoblot, with a different anti-PKR antiserum from that used for immunofluorescence and with anti-actin antisera as a loading control. No PKR was detected in the PKR-TKO MEFs or BMDMs (Fig. 1B and C). We did not detect any smaller products in the PKR-TKO MEFs or BMDMs with this antibody, suggesting that no truncated PKR products were made. Thus, we have confirmed that as expected, the PKR-TKO MEFs do not express detectable PKR.

**Fig 2:**
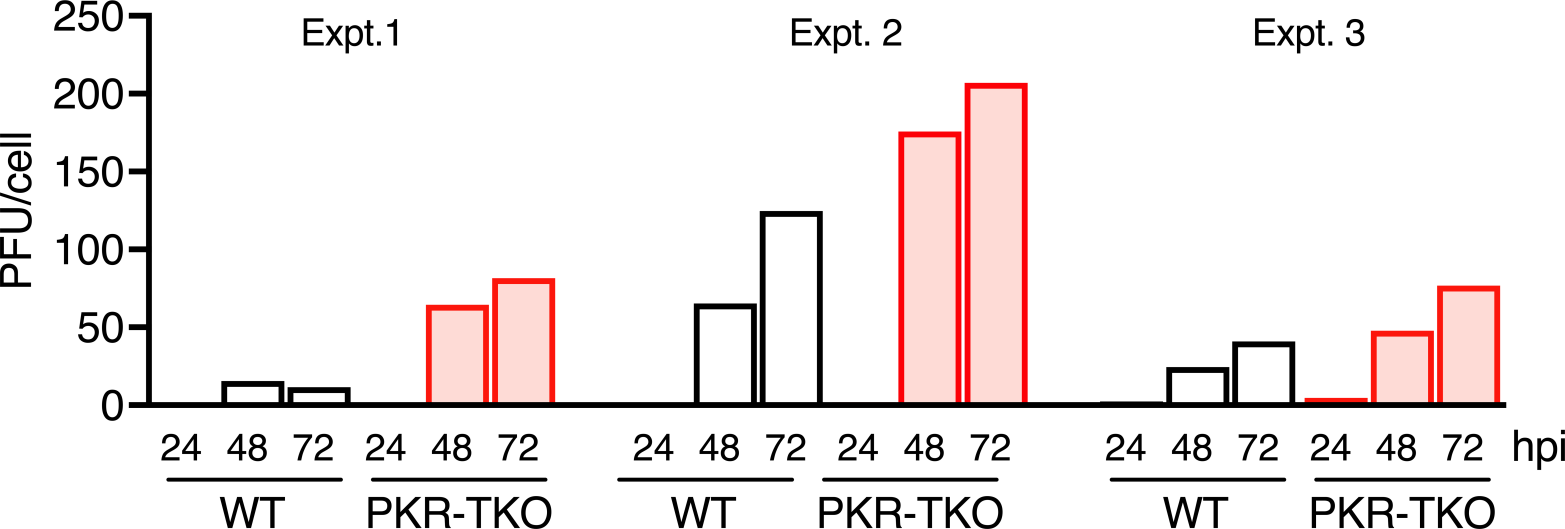
Viral yield in WT and PKR-TKO MEFs. WT and PKR-TKO MEFs were infected with MAV-1 at MOI of 5 and harvested at 24, 48, and 72 hpi. Virus titers were determined by plaque assay in 3T6 cells. Because of possible plating differences between the two cell types, yield (PFU/cell) was determined by multiplying the determined titer of an aliquot (PFU/mL) by the total volume per sample well and dividing by the number of cells counted for each cell type at 48 hpi. MEF infections were performed in biological duplicates at each time point, and the graph shows the average PFU/cell values obtained for each condition. Three independent experiments are shown. Because there was a variation of control sample titers among the plaque assays, for statistical analysis, we performed a Wilcoxon matched-pairs signed rank test, comparing WT to PKR-TKO titers by time point within each experiment. Titers in the PKR-TKO cells were significantly higher (*P* = 0.0039).

We infected WT and PKR-TKO MEFs with WT MAV-1 to determine whether the lack of PKR affected virus yield. Cells were infected at an MOI = 5, harvested at 24, 48, and 72 h post infection (hpi) in their media, lysed, and virus yields were assayed by plaque assay on mouse 3T6 cells. No appreciable virus was detected at 24 hpi (Fig. 2). In three independent experiments, the virus yields from PKR-TKO MEFs were higher than those from WT MEFs, both at 48 and 72 h. This difference is statistically significant (Wilcoxon matched-pairs signed rank test, *P* = 0.0039) and is consistent with MAV-1 infection of other PKR knockout MEF lines (6). This indicates that as expected, PKR is an effective antiviral host protein, reducing viral yield in cultured cells.

### MAV-1 infections of PKR-TKO mice indicate that PKR is antiviral *in vivo*

**Fig 3:**
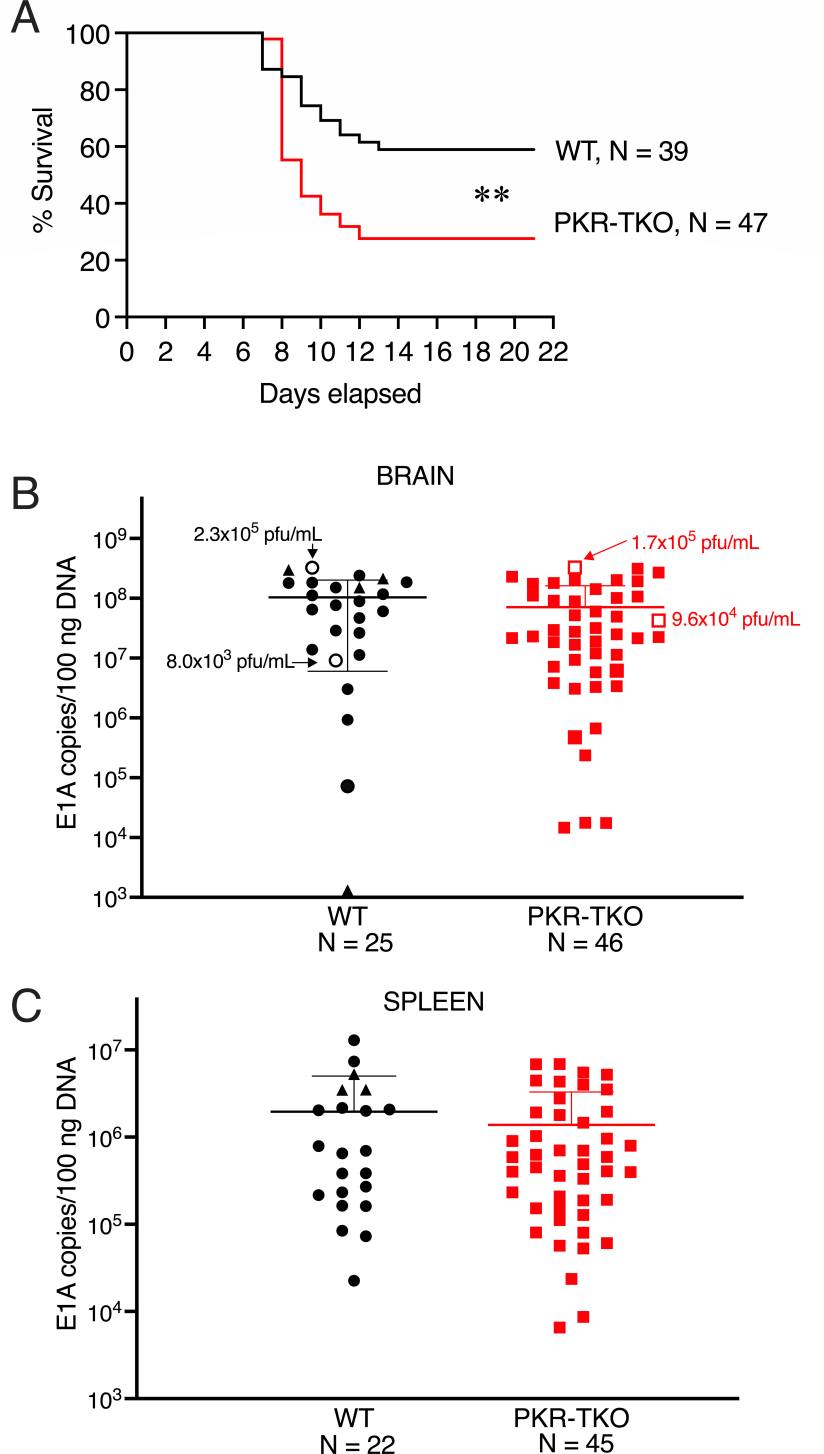
Growth of MAV-1 in WT and PKR-TKO mice. (**A**) Mice were infected i.p. with 10^2^ PFU of MAV-1. Mice were euthanized when they reached a threshold disease score based on clinical signs. *N*, number of mice per condition. Survival of the two strains was statistically significantly different by both the Mantel-Cox test and the Gehan-Breslow-Wilcoxon test (*P* = 0.0041 and *P* = 0.0045, respectively). (**B**) Viral DNA in brains harvested at 8 dpi from mice infected with 10^2^ PFU of MAV-1. Viral DNA copies were determined by qPCR using primers for MAV-1 E1A. For the indicated samples with open symbols, brain homogenates were assayed for infectious virus by plaque assay; titers are shown. Four WT mice indicated by triangles were mice obtained from Jackson Laboratory; all others were bred in-house. (**C**) Viral DNAs in spleens from mice in (**B**) were quantitated by qPCR as for brain samples in (**B**). For B and C, the limit of detection in the qPCR analysis was determined as 10^3^ copies/100 ng DNA based on the standard curve. There was no statistical difference in viral loads in brains or spleens of the two mouse strains.

Given the antiviral nature of PKR, we expected that mice lacking PKR would be more likely to die from MAV-1 infection than WT mice. We assayed survival of WT C57BL/6 mice bred in the same animal room and PKR-TKO mice at two doses of WT MAV-1 infection. At 10^2^ PFU/animal, there was a statistically significant difference in survival: 59% of WT mice survived compared to 28% of the mutant mice (Fig. 3A). At 10^3^ PFU MAV-1/mouse, there was no significant difference between strains and only ~14% of mice survived (data not shown). We calculated an LD_50_ for WT mice of 10^2.2^ PFU, whereas the LD_50_ in PKR-TKO mice was 10^1.5^ PFU (Table 1, Expt. 1). For subsequent experiments investigating parameters that might differ between WT and PKR-TKO mice, we used a dose of 10^2^ PFU/mouse and assayed at 8 dpi, at the peak of cytokine responses in previous studies (24) and just prior to onset of mortality.

**TABLE 1:** LD50 of WT and E4TMC2 viruses

		LD_50_ (PFU)				
Mouse strain	Virus	Expt. 1	Expt. 2	Expt. 3	Expt. 4	Expt. 5
C57BL/6	WT	10^2.2^	10^3.8^	**––**	**––**	**––**
	E4TMC2	–– ^*a*^	>10^6.0^	>10^7.0^	**––**	**––**
SJL/J	WT	**––**	**––**	**––**	10^-0.32 *b*^	**––**
	E4TMC2	**––**	**––**	**––**	>10^7.0^	**––**
PKR-TKO	WT	10^1.5^	**––**	**––**	**––**	**––**
	E4TMC2	**––**	**––**	**––**	**––**	>10^7.0^

^*a*^ ––, not determined.

^*b*^ From references (25, 26).

The highest levels of MAV-1 in infected mice are found in the brain, spinal cord, and spleen (27–29). We assayed viral loads in brains and spleens of WT and PKR-TKO mice infected with 10^2^ PFU/animal, both by quantitating viral DNA by qPCR and by quantitating infectious virus by plaque assays (Fig. 3B and C). Although the survival curve (Fig. 3A) indicates that up to 55% of mutant mice might be dead on day 8, which might alter the analysis of viral loads from only the surviving mice, in the experiments shown in Fig. 3B and C, no mice succumbed prior to day 8. We were surprised to find no significant differences in viral loads in either organ by viral genome quantitation or infectious virus assay. This contrasts with our experience with other mice that are more susceptible to MAV-1 infection than WT mice, in which higher susceptibility correlates with higher brain and spleen viral loads. This includes mice of some susceptible inbred strains (30), mice that are deficient in B cells or Bruton’s tyrosine kinase (31), and mice lacking the interleukin 1 receptor (24). The lack of differences in viral loads between infections of WT and PKR-TKO mice suggested that something other than differences in levels of viral replication was responsible for the reduced survival of PKR-TKO mice infected with MAV-1.

### Comparisons of MAV-1 pathogenesis in WT and PKR-TKO mice

**Fig 4:**
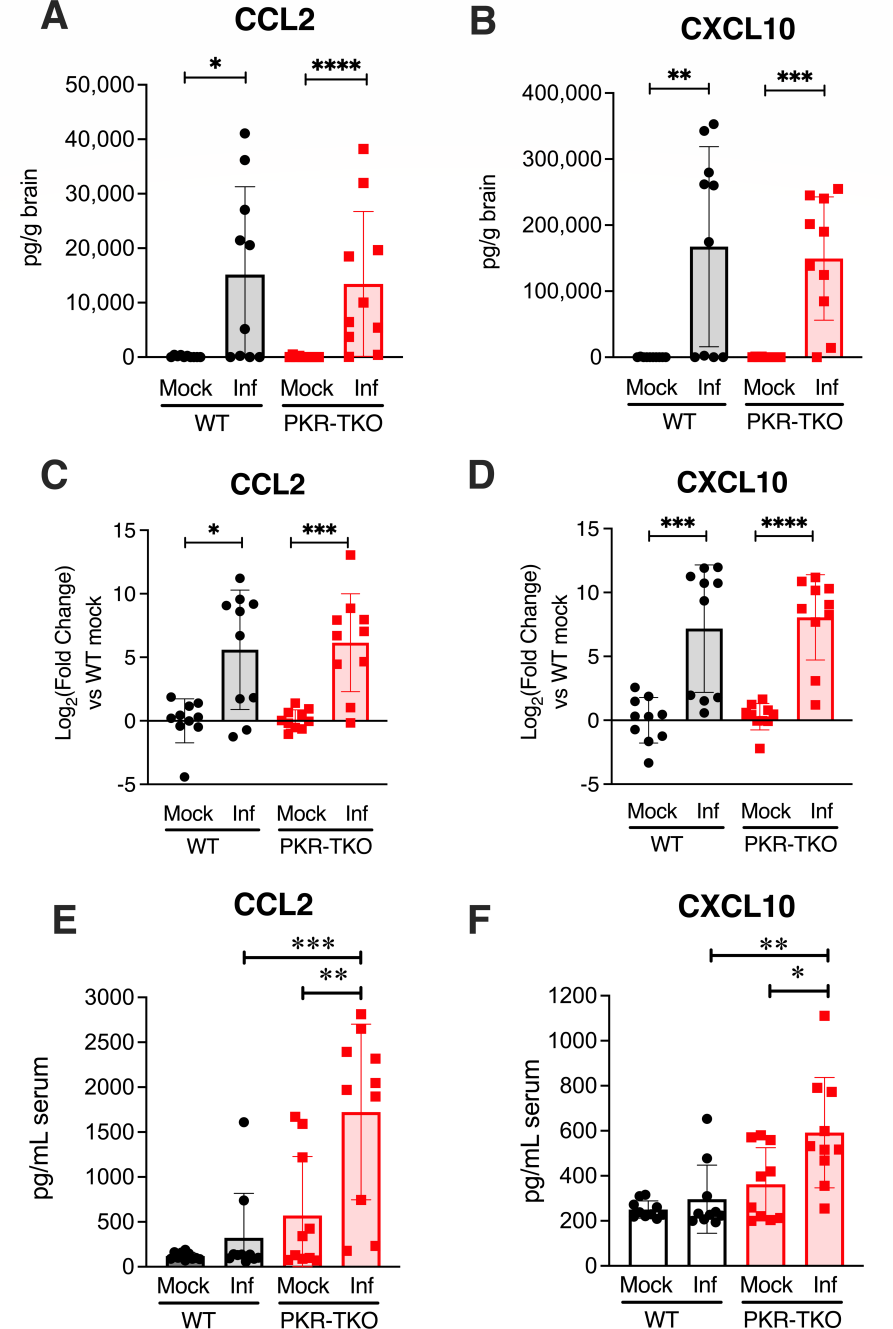
Chemokine analysis of MAV-1-infected brains and serum 8 dpi. CCL2 (**A, C, and E**) and CXCL10 (**B, D, and F**) were assayed by ELISA from technical duplicates of brain homogenates (**A and B**) or serum (**E and F**) of MAV-1- or mock-infected WT and PKR-TKO mice. Data were analyzed by the Mann-Whitney test, comparing mock to infected (Inf) within each strain and by comparing MAV-1-infected strains. (**C and D**). Gene expression in brains of the same chemokines and same mice as in (**A**) was analyzed by RT-qPCR. For each sample, amplified in technical duplicates, ΔCt was normalized to β-actin, and ΔΔCt was normalized to the WT mock group for each chemokine. Statistical analysis was performed using the Mann-Whitney test between groups, and statistical analyses were performed on the log_2_-fold change data (*, *P* < 0.05; **, *P* < 0.01; ***, *P* < 0.001; ****, *P* < 0.0001).

One explanation for the higher mortality in PKR-TKO mice compared to WT mice without accompanying higher viral load is that in the absence of PKR, there may be an altered inflammatory response that contributes to the disease severity. To address this, we assayed cytokine levels in brains and sera of infected mice of both strains (Fig. 4). Mice were infected with 10^2^ PFU of WT virus for 8 days, a time when we previously observed altered chemokine levels in brains (24). The most notable findings were increased levels of CCL2 and CXCL10 pro-inflammatory chemokine protein and mRNA levels in brain homogenates by enzyme-linked immunosorbent assay (ELISA) and reverse transcriptase quantitative PCR (RT-qPCR) assays, respectively, upon infection. However, there were no significant differences for brains between mouse strains; but in sera, CCL2 and CXCL10 levels were higher than WT in the PKR-TKO mice (Fig. 4E and F). We also saw modest protein level increases upon infection for IFNα and IL-6 for both mouse strains (Fig. S1). For IFNβ, IL-1β, IFNγ, and TNFα, there were no significant differences between mock and infected mice. Moreover, for all these cytokines and chemokines, there was no significant difference in protein levels between WT and PKR-TKO mice (Fig. S1). The results suggest that differences in these inflammatory modulators do not explain higher mortality in MAV-1-infected PKR-TKO mice.

**Fig 5:**
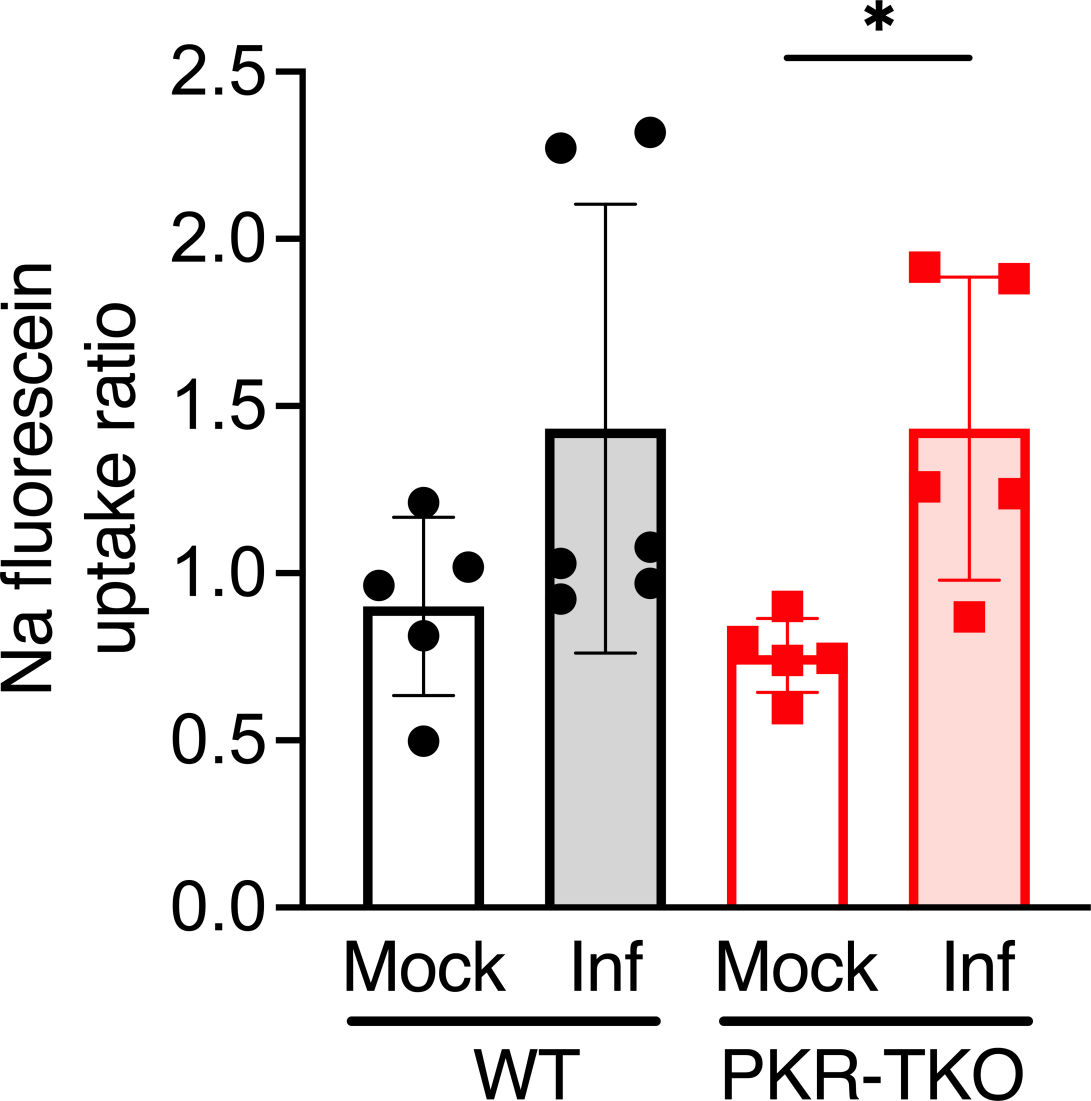
Sodium fluorescein uptake comparison between WT and PKR-TKO mice. Mice were infected for 8 days with 10^2^ PFU of WT MAV-1 (Inf) or mock infected. Ten minutes before euthanasia, sodium fluorescein was injected i.p. Sera and brains were collected and analyzed for sodium fluorescein levels. The ratio of sodium fluorescein in brain relative to serum (uptake ratio) is shown for mock and infected mice and was calculated as described in Materials and Methods. Data were analyzed by Mann-Whitney test, and the only significant difference is indicated (*, *P* < 0.05).

MAV-1 infection results in disruption of the blood-brain barrier, which can be assayed by permeability to sodium fluorescein and increased activity levels of matrix metalloproteinases (MMP-2 and MMP-9) (32, 33). We quantitated brain levels of sodium fluorescein after i.p. injection in WT or PKR-TKO mice infected with 10^2^ PFU of WT virus for 8 days (Fig. 5). Although some infected mice showed increased brain sodium fluorescein levels relative to mock-infected mice, there were no significant differences in brain permeability between WT and PKR-TKO mice. This result is consistent with our findings of no differences in brain viral loads between strains (Fig. 3). In our experience, MAV-1 infections of mice show a correlation between permeability to sodium fluorescein and brain viral loads (24, 32).

### The Cul2-binding domain of MAV-1 E4orf6 is important for PKR degradation in cell culture infections

The E4orf6 protein is required for the degradation of PKR in transfection experiments *in vitro* (7). In MAV-1 infection of cultured cells, PKR degradation is proteasome dependent (6) and is blocked by the inhibition of Cul2 (7), a component of the E3 ubiquitin ligase assembled in MAV-1-infected cells (20). Cul2 binds to MAV-1 E4orf6, and a mutational analysis showed that three hydrophobic amino acids of E4orf6 are required for the binding. Using transfection assays, we showed that a plasmid encoding the E4orf6 protein with all three hydrophobic amino acids mutated results in substantially reduced PKR degradation in conditions where the WT E4or6 leads to complete degradation of PKR (7). We hypothesized that a virus with these three E4orf6 mutations would be unable to induce PKR degradation in cell culture and would have altered pathogenesis in mice.

**Fig 6:**
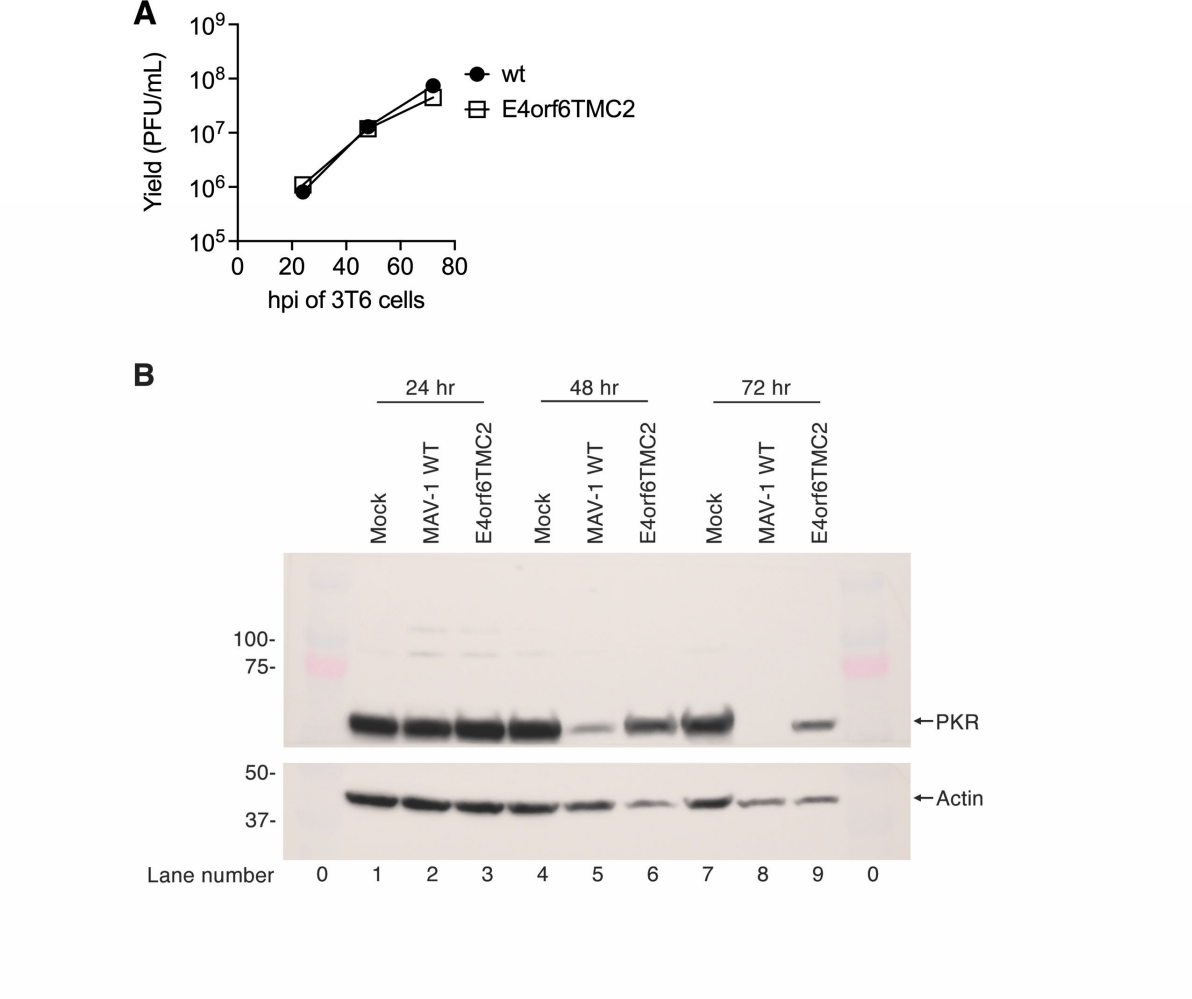
Growth and PKR degradation by WT and E4orf6TMC2 MAV-1. 3T6 cells were infected with WT MAV-1 at MOI 5 and harvested at the indicated times. (**A**) Virus yields were determined by plaque assay on E4I11 cells induced with dexamethasone to express E4orf6. (**B**) Cell extracts were analyzed by immunoblot. Prior to incubating with primary antibodies, the membrane was cut in half horizontally between the positions for the PKR and actin proteins. The top half was incubated with anti-PKR antibody B-10 at 1:350 and the lower half with anti-actin antibody at 1:1,000. Lanes 0 are molecular weight markers whose sizes are shown on the left. The experiment was performed in duplicate; representative virus yields and immunoblot assay results are shown.

To test the importance of PKR degradation in MAV-1 infection of cultured cells and mice, we used recombineering to construct a viral mutant in MAV-1 E4orf6, named E4orf6TMC2, as described in Materials and Methods. MAV-1 E4orf6TMC2 has the three amino acid changes in the E4orf6 protein sequence relative to WT E4orf6 that are important for Cul2 binding and PKR degradation in plasmid transfections (Y69R, V73K, and L77K) (7, 20). The mutated region was confirmed by sequencing a PCR product from the virus stock. Subsequently, the entire genome sequence was confirmed by whole-genome sequencing, verifying that the E4orf6TMC2 virus had the expected changes and no off-target mutations. To characterize the mutant virus in cell culture, we infected mouse 3T6 cells with WT MAV-1 or MAV-1 E4orf6TMC2 at MOI = 5 and harvested at 24, 48, and 72 hpi. Cells were harvested in their media, lysed, and supernatants titrated by plaque assays. The results showed that there was no difference in virus yield between WT and mutant virus (Fig. 6A). We sequenced virus from the 72 hpi samples and confirmed that the E4orf6TMC2 mutations were still present. This result indicates that the three amino acid changes in MAV-1 E4orf6 did not alter the ability of the mutant virus to replicate in cell culture.

From the same infections assayed in Fig. 6A, parallel aliquots were washed and scraped in Dulbecco’s phosphate-buffered saline (DPBS), pelleted, and processed by lysis in Laemmli gel sample buffer (LGSB) for immunoblot analysis. Levels of PKR were determined by incubation with an anti-PKR antibody, and antibody to actin was used as a control (Fig. 6B). The results show that PKR was completely degraded by 72 hpi in the WT MAV-1 virus infection (lane 8), as we have previously shown (7). This contrasts with less degradation in cells infected with the mutant virus MAV-1 E4orf6TMC2 (lane 9). This result indicates that the domain of the E4orf6 protein involved in Cul2 binding that is mutated in the E4orf6TMC2 virus is important for PKR degradation in virus infection in cell culture. This is consistent with the results previously obtained in plasmid transfections with plasmids expressing E4orf6 with the same three amino acids changed (7). The fact that some degradation was evident in the E4orf6TMC2 virus infection suggests that the amino acid changes in E4orf6 that are involved with Cul2 binding were not sufficient to completely block PKR degradation.

### The Cul2-binding domain of MAV-1 E4orf6 is important for virus lethality in mice

We then infected mice with WT MAV-1 and the E4orf6TMC2 mutant virus. We performed an LD_50_ analysis, infecting groups of 5 or more mice at doses from 10^2^ to 6.25 × 10^6^ PFU (Table 1, Expt. 2). The LD_50_ of WT virus in C57BL/6 mice was 10^3.8^ PFU, whereas the LD_50_ of the mutant virus was >10^6.0^ PFU. At the highest dose of the mutant virus we could administer, no mice succumbed to infection (Table 1, Expt. 3). This result suggests that the function of E4orf6 altered in the mutant virus is essential for MAV-1 lethality in C57BL/6 mice even though there is incomplete PKR degradation. Because C57BL/6 mice are relatively resistant to MAV-1, we tested a more susceptible strain, SJL/J (25). SJL/J mice also did not succumb to infection by E4orf6TMC2, although at 8 dpi, two mice of five infected with the maximum dose (1.1 × 10^7^ PFU) showed mild signs of disease (lethargy) but recovered within 12–18 h. The LD_50_ in SJL/J mice was thus >10^7^ PFU, considerably higher than that of WT virus (10^-0.32^ PFU; Table 1, Expt. 4). We tested E4orf6TMC2 infection of PKR-TKO mice, and no mice succumbed at the highest dose; the LD_50_ was >10^7^ PFU (Table 1, Expt. 5). We confirmed that all three strains of mice (C57BL/6, SJL/J, and PKR-TKO) had been infected by the mutant virus, using ELISA to measure anti-MAV-1 IgM in serum at 21 dpi (34). Sera from both WT and mutant virus-infected mice were positive (data not shown), indicating that though the E4orf6TMC2-infected mice survived, they had received virus and developed antibodies. Taken together, the results indicate that mutation of E4orf6 substantially reduced MAV-1 virulence in mice.

**Fig 7:**
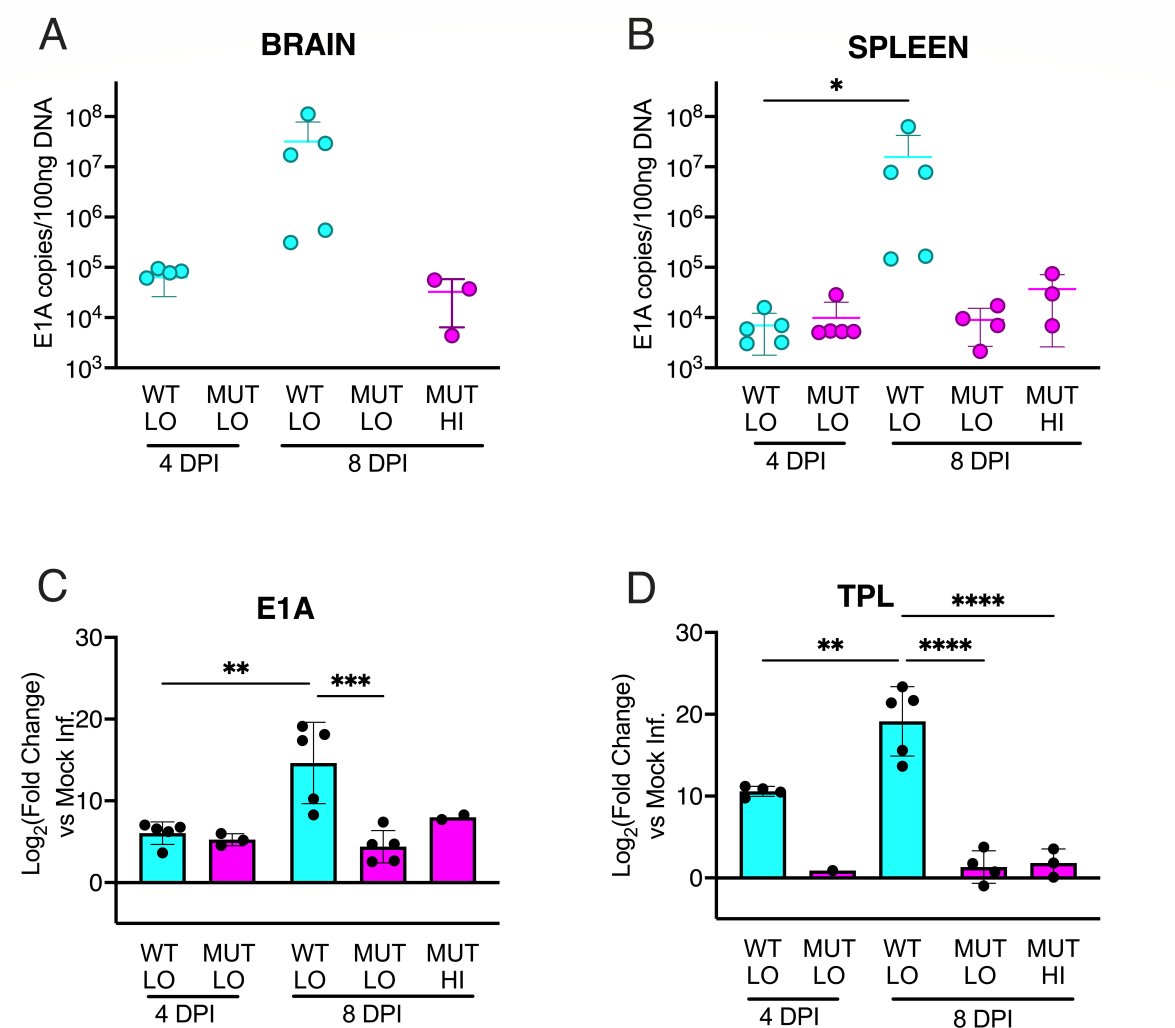
Viral replication and gene expression. WT or E4orf6TMC2 (MUT) viruses were used to infect C57BL/6 mice i.p. for 4 or 8 days. Groups of five mice were infected at 10^2^ PFU low (LO) for 4 or 8 days, or 10^7^ PFU high (HI) for 8 days, and a group of three mice was mock infected. Only the mutant virus was given at the high dose. (**A and B**) Homogenates of brains or spleens were prepared and assayed for viral DNA. The limit of detection was determined as 10^3^ copies/100 ng DNA based on the qPCR standards. Many of the sample values were below the limit of detection and thus are not depicted. The mean and SD are shown when ≥3 samples had detectable MAV-1 viral genome loads. (**C and D**) Brain homogenates from the same mice used in (**A**) were used to prepare RNA. The RNAs were analyzed by RT-qPCR for expression of mRNA for early protein E1A (E1A) and for expression of mRNA for late proteins whose mRNAs have the tripartite leader (TPL). Mean and SDs are shown. Statistics were analyzed as in Fig. 4 with normalization to β-actin, and fold changes were normalized to mock. Analyses were performed on the sample values rather than fold change. Statistical significance was determined by one-way analysis of variance (ANOVA) with Tukey’s multiple comparisons tests (*, *P* < 0.05; **, *P* < 0.01; ***, *P* < 0.001; ****, *P* < 0.0001).

We determined whether C57BL/6 mice infected with the E4orf6 mutant virus had altered viral loads compared with WT virus that would indicate differences in viral replication *in vivo*. Groups of five mice were infected with 10^2^ PFU of WT or mutant virus for 4 or 8 days. A group of 5 mice was infected with the maximum dose of the mutant virus we could give, 10^7^ PFU, for 8 days. Only the mutant virus was given at the high dose. Brain and spleen homogenates were assayed for viral DNA using qPCR. At 4 dpi, only low levels of the WT virus were detected in brains and spleens (Fig. 7A and B). However, at 8 dpi, high levels of virus were found in WT virus infections of brains and spleens, consistent with previously published results for MAV-1 infections, in which organ virus loads increase over time of infection (25, 35). In contrast, at the low virus dose, little to no MAV-1 DNA was found in brains or spleens of mice infected with the mutant virus at either time point. At the highest dose possible, the mutant viral DNAs were detected in a few mice at low levels that were similar to those of the WT virus at 4 dpi. This strongly suggests that the mutant virus is unable to replicate to high levels in mice.

We examined levels of viral early and late mRNAs by RT-qPCR from the same mouse brains as in Fig. 7A. Both early and late viral gene expression increased between 4 and 8 dpi for WT-infected mice (Fig. 7C and D). In contrast, there was very little early or late viral gene expression in brains of mice infected with the low or high dose of the E4orf6TMC2 virus. This is consistent with the viral load results. Taken together, the results indicate that the mutant virus is deficient in early gene expression, viral DNA replication, and late gene expression.

**Fig 8:**
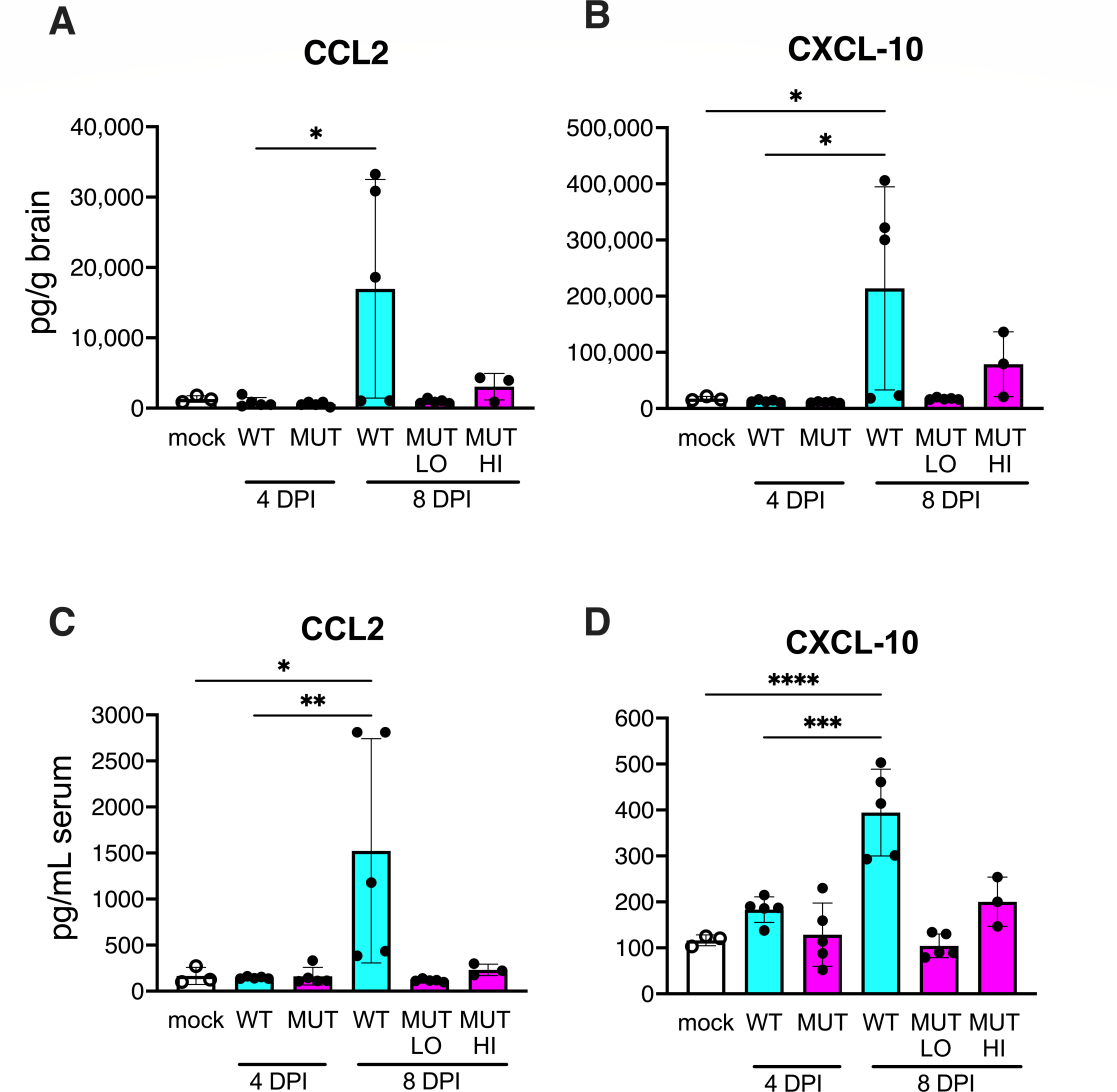
Chemokine analysis of E4orf6TMC2 mutant virus-infected brains. The same C57BL/6 mice in Fig. 7 that were infected i.p. with low or high doses of WT or E4orf6TMC2 (MUT) viruses were analyzed for chemokines by ELISA assay. The mean and SD are shown. (**A and C**) CCL2 and (**B and D**) CXCL10 were assayed from technical duplicates of brain homogenates (**A and B**) or serum (**C and D**). Statistical significance was determined by one-way ANOVA, and the significant results of Tukey’s multiple comparison tests are shown for comparisons by virus and by virus compared to mock infection (*, *P* < 0.05; **, *P* < 0.01; ***, *P* < 0.001; ****, *P* < 0.0001).

We examined levels of chemokine and cytokine proteins in infections of C57BL/6 mice with WT and E4orf6TMC2 virus. For both viruses, we observed higher levels of CCL2 and CXCL-10 compared to uninfected mice (Fig. 8). However, the levels were higher for WT virus infections than for the mutant virus levels, which were only marginally higher than for mock infections, even for the high dose mutant infections at 8 dpi. These results correlate with the lower mutant virus replication and gene expression compared to WT virus (Fig. 7). Additional cytokines were also assayed by ELISA (IFNα, IL-6, IFNβ, IL-10, IFNγ, and TNFα) (Fig. S2) but did not show differences that could explain differences in WT vs mutant viral growth or gene expression.

Finally, we examined histology in mice infected with the mutant virus. Four C57BL/6 mice were infected with WT or E4orf6TMC2 virus at the low dose (10^2^ PFU), or mock infected; and two mice were infected at the high dose (10^7^ PFU). Tissues were examined at 8 dpi by hematoxylin and eosin staining. Mice infected by low dose of WT or E4orf6TMC2 virus did not show any brain lesions (Fig. S3B and D). However, at low dose, both viruses caused liver lesions of similar severity and distribution, consisting of mild multifocal hepatitis. No other lesions were present in the heart, kidney, thymus, lung, and spleen, and no lesions were present in any of the organs from mock-infected mice. This is not consistent with the higher level of CCL2 and CXCL10 in brains of WT-infected mice (Fig. 8). At the high dose of mutant virus, mice showed multifocal microhemorrhages throughout the brain parenchyma (Fig. S3C), similar to brain findings in MAV-1 infections of susceptible and immunodeficient mice infected with ≥7 × 10^2^ PFU (24, 31, 35, 36). The livers of the high-dose E4orf6TMC2-infected mice showed multifocal hepatitis with occasional hepatocellular degeneration and necrosis. Also, the high dose of mutant virus resulted in diffuse myocarditis with scattered microhemorrhages throughout the myocardium and piecemeal myocardial degeneration (Fig. S3G). The increased histopathology in the mice receiving the higher dose of mutant virus was not accompanied by increased lethality: 0/5 mice in our LD_50_ experiment died at the high dose infection out to 21 dpi. The fact that there is increased histopathology with the higher dose of the mutant virus compared to a low dose without increased lethality of the virus suggests that the ability of mice to clear the mutant virus is not affected by tissue damage to the brain, heart, and liver that it may cause.

## DISCUSSION

PKR is an effective antiviral gene product in MAV-1 infections; in its absence, virus yields in cell culture are increased (Fig. 2, this work; 6), and mice lacking PKR (PKR-TKO) are more susceptible to MAV-1 infection (Fig. 3). However, despite higher mortality and a lower LD_50_ for MAV-1 infection of PKR-TKO mice compared to WT mice, there were no differences between strains in brain or spleen viral loads, no differences in blood-brain barrier disruption, and only one difference in the many cytokines assayed (serum levels of CCL2 and CXCL10). It is possible that this difference in systemic CCL2 and CXCL10 contributes to higher mortality of the PKR-TKO mice. We did not assay for liver damage in these mice because it has previously been uninformative; hepatitis was later noted for infection of WT mice by the mutant virus, and it possibly is a factor in mortality of the PKR-TKO mice. Further experiments will address these possibilities. It is possible that a pathway of PKR antiviral function not measured by our assays is responsible for the higher growth of MAV-1 in the PKR-TKO MEFs compared to WT MEFs. For example, PKR induces apoptosis in response to infection by viruses including poxviruses, influenza virus, and picornaviruses (37–40), in a manner that can be dependent on or independent of eIF2α phosphorylation (reviewed in reference 4). Also, PKR is involved in innate immune signaling. Mice deficient in PKR have altered responses to Toll-like receptor (TLR) ligands; PKR involvement is implicated in cascades triggered by TLRs 3, 4, and 9 (reviewed in ref. 41). We have not investigated apoptosis in MAV-1 infection of PKR-TKO vs WT mice. However, we assayed immune signaling pathways in brains of the two mouse strains by ELISA, and there was no difference in levels of interferon (IFN) (Fig. S1). Future studies of apoptosis and innate immune signaling will help elucidate the antiviral nature of PKR in MAV-1 viral infection.

The mechanism by which PKR is induced by adenovirus infection has long been postulated to be through production of dsRNA by annealing of transcripts arising from opposing strands of the adenovirus genome (42). However, support for this is minimal. One study reported production of dsRNA 7 hpi with HAdV5, detected by the J2 monoclonal antibody that recognizes dsRNAs longer than 40 bp (43). Another reported a weak positive J2 staining signal from an adenovirus infection (44). However, these findings are contradicted by recent studies that suggest that WT virus infection does not produce dsRNA (42). Price et al. used J2 and a second more sensitive antibody to dsRNA, 9D5. They examined HAdV 5-infected A549 human lung cells at 24 and 48 hpi but found no evidence of dsRNA staining in multiple images. They also saw no dsRNA staining in infection by an adenovirus mutant lacking VA RNA. However, Price et al. (42) did detect nuclear dsRNA in cells infected with an E4 deletion virus, but only in the subset of cells that are in the late stage of infection. Using mutant viruses in E4orf6 and E1B 55K, the authors showed that both HAdV5 E4orf6 and E1B 55K are required to prevent dsRNA formation during viral infection, and this nuclear dsRNA is enriched for viral unspliced transcripts. These findings explain the appearance of dsRNA in infections by mutants in E4orf6 and E1B55K, but they beg the question as to what induces PKR activity in a WT virus infection by HAdV or MAV-1, and why PKR must be overcome (by VA RNAs for HAdVs and by induction of degradation in the case of MAV-1).

Binding of PKR to dsRNA leads to PKR activation, enabling its dimerization and autophosphorylation; activation is regulated by a dsRNA-binding domain of PKR (45, 46). However, binding to dsRNA is not essential for activation; other mechanisms are possible, particularly in the absence of viral infection. For example, high concentrations of PKR (which causes it to dimerize) and heparin can lead to PKR activation in the absence of dsRNA (47, 48). Also, cellular protein activator of PKR (PACT) is important for PKR activation in the absence of dsRNA binding, and when PACT is overexpressed, there is phosphorylation of PKR and eIF2α and translation inhibition (49). Endoplasmic reticulum (ER) stress is also indirectly responsible for PKR activation. ER stress induces PACT, leading to the activation of pre-existing PKR and contributing to apoptosis (50). Another mechanism of PKR activation independent of dsRNA is through metabolism. Activation of PKR occurs in situations of excess nutrients and obese mice (51, 52). Finally, it has recently been proposed that ssRNA can also activate PKR (53), but the ssRNA examples cited all have some secondary structure except for polyU, polyC, and polyA (53, 54). Although dsRNA has been undetectable in rigorous assays of WT HAdV infections (42), it is formally possible that very low dsRNA levels that could activate PKR are indeed present. Alternatively, one of the above mechanisms or another as-yet-unidentified one may be the trigger for PKR activation in adenovirus infections.

MAV-1 counteracts the effects of PKR by inducing degradation of the protein via the proteasome (6). Previous work *in vitro* showed that the MAV-1 E4orf6 protein induces PKR degradation, and this is dependent on an interaction between E4orf6 and the cellular protein Cul2, part of a ubiquitin ligase complex involved in proteasomal degradation (7). A domain of E4orf6 involved in binding Cul2 is required for PKR degradation, as shown in transfection experiments where three amino acid changes were introduced into an E4orf6 plasmid. Compared to the WT E4orf6 plasmid, the mutated E4orf6 plasmid resulted in reduced PKR degradation. To confirm those transfection results, in this report, we constructed a virus with the same three amino acid changes in E4orf6, so we could test the importance of Cul2 binding in infected cells and mice. In infection of mouse 3T6 fibroblasts in culture (Fig. 6A), the mutant virus produced equal virus yields compared to WT virus, indicating that the E4orf6 mutations were not consequential for virus growth *in vitro*. However, in the same experiment, mutation of E4orf6 in the virus impaired PKR degradation in 3T6 cells relative to WT virus. This is consistent with the previous results with the mutated E4orf6 in plasmid transfection (7) and indicates that the E4orf6-Cul2 interaction is necessary for PKR degradation also in virus-infected cells *in vitro*. However, changing the three amino acids was not sufficient to remove all PKR degradation during infection, as seen in Fig. 6B. One explanation is that the E4orf6 mutant is leaky in mice, that is, the three amino acid changes do not abolish all PKR degradation activity. Alternatively, it is possible that there are other aspects of E4orf6 that contribute to the induction of PKR degradation. Perhaps the entire domain encompassing the three changed amino acids or other regions of E4orf6 contribute to the protein’s function. We attempted to create a virus with E4orf6 deleted, and although we obtained a bacmid with the deletion, we were unable to rescue infectious virus. This suggests that E4orf6 may have other functions critical for infection.

Despite the lack of effect of the E4orf6 mutation in the Cul2 binding site on virus growth in cell culture, the mutation rendered the virus unable to kill mice, even at the highest dose we could administer (Table 1). This was true for C57BL/6 mice, SJL/J mice, and PKR-TKO mice. This E4orf6TMC2 virus, with an LD_50_ > 10^7^ PFU, is thus the most debilitated for mouse pathogenesis of any MAV-1 mutant viruses we have made, including E1A and E3 mutant viruses (34, 55). Compared to WT virus, there was less mutant viral replication in the brain and spleen of infected C57BL/6 mice at 8 dpi, even at a 100,000-fold higher dose (Fig. 7). Early and late viral gene expression were also reduced for the mutant virus. This result indicates that the E4orf6 function that involves interaction with Cul2 is essential for virus replication in mice, even mice lacking PKR, the PKR-TKO mice. Because of this latter finding, the results suggest that the E4orf6 function required for mouse virulence is not only to induce degradation of PKR. A situation similar to that of the MAV-1 E4orf6 mutation occurs in vaccinia virus (VV), in which the N-terminal domain of viral E3L, a PKR inhibitor, is dispensable in cell culture but not in mice (56). This finding correlates with the identification of a function in the VV E3L N-terminus that is not required for inhibition of PKR.

Examination of histopathology induced by the E4orf6TMC2 mutant virus indicated that there were no differences between WT and the mutant virus at the low dose (10^2^ PFU). We have previously noted brain lesions in C57BL/6 mice infected with 700 PFU of virus (31, 36) and were surprised that there was no brain pathology seen here at 10^2^ PFU, although there were accompanying increases in brain and serum levels of chemokines CCL2 and CXCL10. It is possible that there is a specific dose threshold for tissue damage effects to be observed. At the 10^7^ PFU dose of the mutant virus, we saw brain pathology as previously seen with higher doses of WT MAV-1 (24, 31, 35, 36). The high-dose mutant virus also resulted in myocarditis and liver damage, which have also been reported for MAV-1 infections (31, 57–59). The decreased levels of induced cytokines and the avirulence of the mutant virus when administered at high dose, despite signs of tissue damage, indicate that there are other factors affecting mouse survival that should be explored in this system.

What other functions of MAV-1 E4orf6 might be involved in mouse virulence? It is possible that degradation of other cellular proteins besides PKR is involved. The HAdV E4orf6 protein, with or without E1B 55K protein, leads to the degradation of multiple cellular proteins involved in the DNA damage response, whose inactivation provides advantages for virus infection (reviewed in reference 17). In addition, the HAdV E1B 55K and E4orf6 proteins block phosphorylation of PKR and its target eIF2α during infection; this activity is independent of levels of the HAdV PKR inhibitors VA-I and VA-II RNA (60). This phosphorylation of PKR and eIF2α requires the E3 ubiquitin ligase activity mediated by Cul5. One possibility is that HAdV E1B 55K/E4orf6 may lead to degradation of human PKR (61), but in the presence of VA-I or VA-II RNA, such degradation activity is largely masked. This interaction of HAdV E1B 55K/E4orf6 with PKR extends the parallels seen between these proteins in HAdV and MAV-1. Similar to hAdV degradation of cellular antiviral proteins, MAV-1 E4orf6 activity leads to degradation of PKR (7; this work) and DNA ligase IV (20), and together with E1B 55K, MAV-1 E4orf6 triggers degradation of mouse p53 (20). Another function of HAdV E4orf6, in conjunction with E1B 55K as the ubiquitin ligase complex, that might be involved in mouse virulence, is efficiency of viral RNA splicing. Together, the presence of HAdV E4orf6 and E1B 55K results in ubiquitination (without degradation) of two host RNA-binding proteins, relieving viral RNA processing (62). In the absence of the functional E4orf6/E1B 55K complex, late viral unspliced transcripts accumulate in the nucleus. Future work should address these possible sources of virulence due to MAV-1 E4orf6 protein.

Previous studies of viral infections in PKR-deficient mice have used “knockout” mice (21, 22) that were subsequently shown to make a truncated PKR (23). With few exceptions, experiments with these knockout mice have shown modest or no increased susceptibility to viral infection (21, 22, 63, 64), possibly due to the production of truncated PKRs and/or redundant functions with other eIF2α kinases. The use of CRISPR/Cas9 technology to create a PKR knockout mouse in this study resulted in a mouse strain, PKR-TKO, that had significantly reduced survival compared to WT mice when infected with MAV-1 (Fig. 3). PKR-TKO MEFs had increased viral yield compared to WT MEFs (Fig. 2). Moreover, by immunofluorescence and immunoblotting, MEFs from PKR-TKO mice produced no detectable PKR using two antisera (Fig. 1). The CRISPR/Cas9 mutation deletes mouse PKR exon 5 and surrounding splicing regions (7). It is formally possible that a truncated PKR is made that is not detected by the PKR antibodies we used. For immunoblotting, the monoclonal antibody, sc-5282 (B-10), was raised against the entire mouse PKR, but we are unaware of experiments mapping the PKR epitope(s) it recognizes that would enable determining whether it could detect a truncated product from PKR-TKO cells, if made. The rabbit recombinant monoclonal antibody we used in immunofluorescence was raised against a proprietary immunogen (Abcam 184257). While we cannot rule out the presence of truncated PKR products being made in the PKR-TKO knockout cells, our data indicate that full-length PKR is not made. The PKR-TKO mice and MEFs thus provide a good system for study of requirements of PKR for viral infection.

In generating the E4orf6TMC2 mutant virus, we used two E4 complementing cell lines (CCLs) that we constructed. The mutant virus was initially isolated in E4I5 cells, a CCL that could constitutively express the entire E4 region in which we had inserted a 6 nt Kozak recognition sequence in two positions upstream of the E4 protein ATGs. Subsequently, the virus was propagated and titrated in a second E4 CCL, E4I11 cells, which had the WT E4 sequence under the control of an inducible promoter. Both cell types supported propagation of virus. We only used the E4I11 cells for titration of the mutant virus, and we always induced with dexamethasone to obtain E4 expression. We obtained high titer mutant virus stock titers (1.1 × 10^8^ PFU/mL). The titers of WT virus in parallel (1.5 × 10^8^ PFU/mL) were consistently at least 10-fold higher than WT titers on parental 3T6 mouse fibroblasts. This observation suggests that providing cells with additional E4 at the time of MAV-1 infection can increase virus yield, perhaps because of induction of degradation of cellular antiviral proteins including but not only PKR.

### Summary

**Fig 9:**
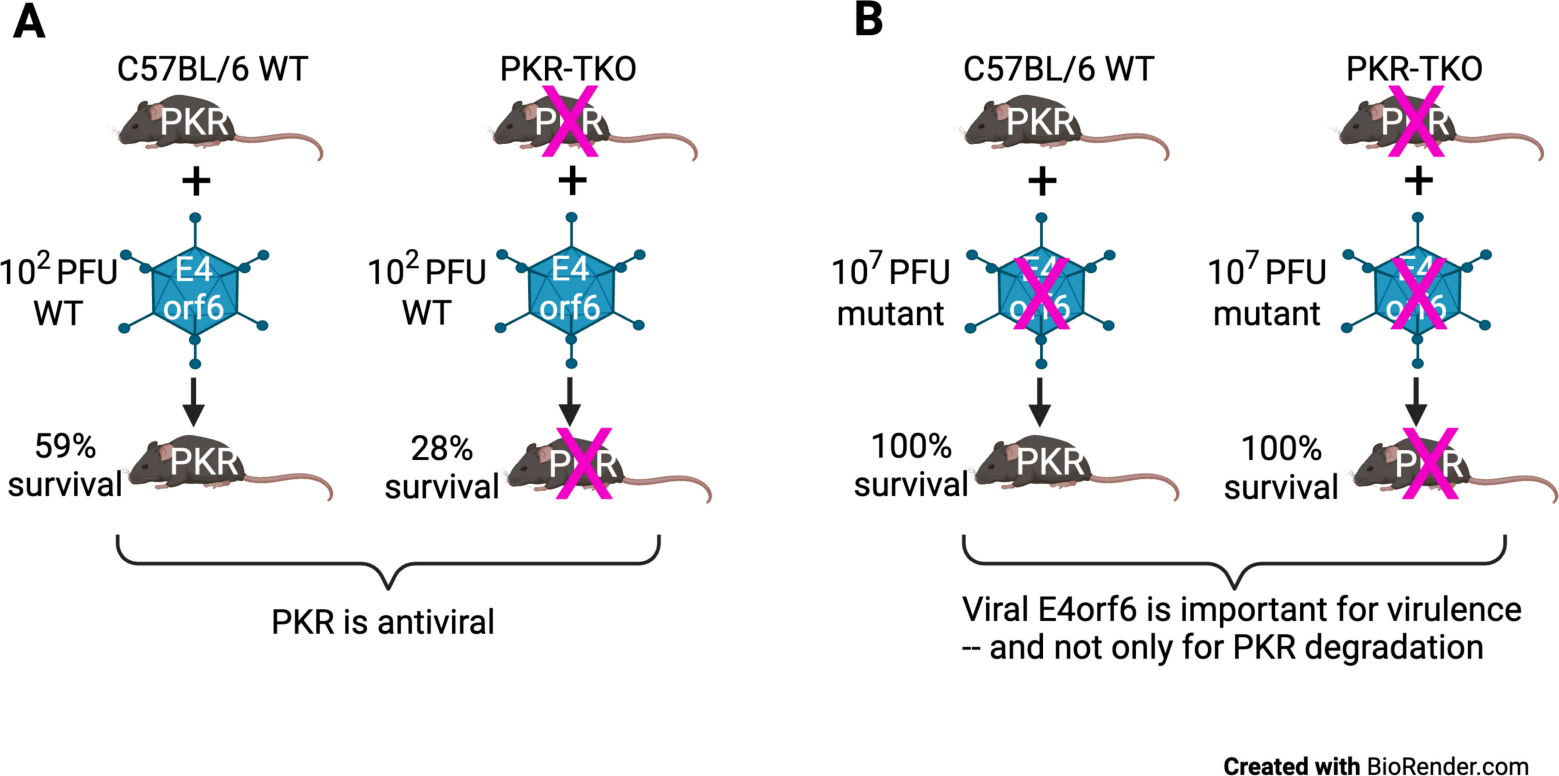
Summary of results. (**A**) Comparison of survival of WT C57BL/6 mice and PKR-TKO mice upon infection with WT MAV-1 at ~1 LD_50_ (10^2^ PFU). “X” indicates knockout of PKR in the PKR-TKO mice. Data based on Fig. 3 and Table 1. (**B**) Comparison of survival of WT and PKR-TKO mice upon infection by mutant E4orf6TMC2 virus at the maximum dose, 10^7^ PFU. The E4orf6 change of three amino acids in the Cul2-binding site is indicated by the “X” on the mutant virus. Data based on Fig. 7 and Table 1.

Figure 9 describes the results of our findings and their implications graphically. Based on our studies with the PKR-TKO cells and mice, we conclude that PKR is an important host antiviral protein that protects cells infected by MAV-1 (Fig. 9A). In the absence of PKR, WT virus infection results in higher mortality, indicating that PKR is antiviral *in vivo* through a mechanism that has yet to be determined. In Fig. 9B, we diagram the results of infections with the E4orf6TMC2 virus, which has alterations in three amino acids of the E4orf6-binding site. This change renders the E4orf6 protein nonfunctional with respect to virulence in both WT mice and mice knocked out for PKR. This indicates that E4orf6 is required for virulence, regardless of whether PKR is present. Since E4orf6 is required for virulence in the absence of PKR, the results support a model in which other functions of E4orf6, as discussed above, are important for MAV-1 pathogenesis.

## MATERIALS AND METHODS

### Cells

All cells were incubated at 37°C in 5% CO_2_. The mouse 3T6 cell line (ATCC CCL-96) was originally obtained from Dr. Alvin Winters, University of Alabama, Tuscaloosa, and cells were maintained in Dulbecco’s modified Eagle’s media (DMEM) containing 5% heat-inactivated newborn bovine serum (HINS). PKR-TKO MEFs were derived from PKR-TKO 15-day mouse embryos as described (7); PKR-TKO transgenic mice lack exon 5 and flanking splicing regions of the mouse PKR gene. C57BL/6 MEFs (B6 MEFs) were derived from WT C57BL/6 littermates obtained during the intercrossing used to obtain the PKR-TKO mice. The B6 MEFs were prepared from 15-day embryos as described for the PKR-TKO MEFs. PKR-TKO and B6 MEFS were maintained in DMEM containing 10% fetal bovine serum. BMDMs were obtained from 8- to 12-week-old B6 or PKR-TKO mice by flushing femurs and tibias with DMEM. They were then differentiated in DMEM containing 20% heat-inactivated fetal bovine serum, 30% L-929 cell-conditioned media prepared in-house (65), 2 mM L-glutamine (Sigma G3126), 1 mM penicillin/streptomcyin (Gibco 15140–122), 1 mM sodium pyruvate (Gibco 11360–070), and 2 mM nonessential amino acids (Gibco 11140–050). After 7 days, cells were seeded for the experiment.

The E4 complementing cell line E4I5 was derived from mouse 3T6 cells, has the MAV-1 E4 gene region, and was prepared as follows. We had a plasmid synthesized by GenScript (Piscataway, NJ). The MAV-1 E4 region corresponds to 29,801–27,188 of NCBI Reference Sequence: NC_000942.1, with an NheI recognition site added at 29,801 and an XbaI recognition site added at 27,281 for insertion into the pcDNA3.1(+) vector. In two places, we had a 6 nt Kozak sequence inserted immediately upstream of the ATG (GCCACC): at 29,711 and 29,277 to optimize translation. This plasmid was named E4pcDNA3.1Kozak, and we transfected 4 μg into 7.5 × 10^4^ 3T6 cells (~50% confluent) with 10 μL of Lipofectamine 2000 (Invitrogen 11668027) in 500 μL of Optimem (Gibco 31985–070) per the manufacturer’s instructions. At 6 h post transfection, we changed the medium to DMEM + 5% HINS. At 2 days post transfection (dpt), we expanded the transfected cells to 150 mm dishes and added 400 μg/mL geneticin (G418; Gibco 10131–035) for selection of transfected cells. At 14 dpt, we picked single colonies by toothpick into wells of 24-well plates containing 1 mL media with 200 μg/mL G418. We expanded the cells over 6–8 weeks until we had enough to confirm E4orf6 protein expression. One line was used for the experiments here, designated E4I5. It was maintained in DMEM containing 5% HINS and 200 μg/mL G418.

The inducible E4I11 cells were similarly derived from 3T6 cells. MAV-1 E4 sequence (from 29,762 to 27,279, NCBI Reference Sequence: NC_000942; the HindIII-E fragment) was cloned into the dexamethasone-inducible expression plasmid pJ5Ω (66) that had been digested with HindIII and treated with calf intestinal phosphatase. Orientation was confirmed by BamHI digest, and the resulting plasmid was named pJ5E4Twist10. We co-transfected 0.1 μg pcDneo (67) and 4 μg pJ5E4Twist10 into 7.5 × 10^4^ 3T6 cells (~50% confluent) with 5 μL of Lipofectamine 2000 (Invtrogen 11668027) in 500 μL of Optimem (Gibco 31985–070) per the manufacturer’s instructions. Cells were expanded, and single colonies were picked as described above for E4I5 cells. One line was used for the experiments here, designated E4I11 cells. For transfection and infection experiments of E4I11 cells, 10^−5^ M dexamethasone (Sigma D4902) in 95% ethanol was added to cells 24 h prior to the infection to induce E4 expression from the mouse mammary tumor virus promoter derived from pJ5Ω (66).

### Mice

All animal work complied with relevant federal and University of Michigan policies. Animals were housed in microisolator cages and provided food and water *ad libitum*. Male C57BL/6 mice (3–5 weeks old) were obtained from Jackson Laboratory (Bar Harbor, ME). We used males because our previous pathogenesis studies used males. We also used C57BL/6 mice of both sexes bred in the same animal room in-house. We observed no significant differences between sexes in clinical signs or other responses to infection. Mice were infected i.p. with the indicated virus dose in 0.1 mL, diluted in DPBS (Corning 21–031-CV). Mock-infected mice were inoculated with conditioned media, which consisted of harvested supernatant from uninfected cells, freeze-thawed three times, and clarified by centrifugation, and diluted in DPBS like the virus for inoculation.

### Virus and infections

WT MAV-1 stock was prepared and titrated on 3T6 cells (68). We prepared the mutant virus MAV-1 E4orf6TMC2 by bacterial artificial chromosome (BAC) recombineering using a bacmid with the full-length MAV-1, pKBS2.MAV1WT(colony16) as the starting material (69). We introduced the mutant E4 sequence into the MAV-1 genome bacmid using GalK selection (70) as follows.

We first introduced GalK into the MAV-1 bacmid, replacing the MAV-1 E4 region (70). To do this, we amplified the GalK gene with a forward primer (TMGALFOR, 5'AAACTTTTGCACAACCAGCTCCCTAACCTTCGCCGCTGCCAAACACTGAAcctgttgacaattaatcatcggca3') containing 50 nt of MAV-1 sequence (upper case, nt 27,296–27,845) followed by 24 nt of GalK cassette sequence (lower case) and a reverse primer (TMGALREV) containing 50 nt of MAV-1 sequence (nt 28,059–28,010) followed by 20 nt of GalK cassette sequence. We digested the amplified mixture with DpnI to remove methylated plasmid template and purified the PCR product. We introduced this PCR product by recombineering into *Escherichia coli* SW102 containing pKBS2.MAV1WT(colony 16) using Recombineering Protocol 3 (https://frederick.cancer.gov/resources/repositories/Brb/#/protocol and reference [71]). We identified GalK+ colonies with the bacmid with the GalK gene replacing the E4 region of MAV-1 and used one (pKSB2.MAV-1colony16Δorf6TM::galK) for the replacement substitution step.

To obtain the DNA fragment to use to replace the GalK with the mutant E4orf6 region, we used plasmid FLAGmE4orf6TMWT8, described previously (7). This plasmid has three amino acid changes from WT E4orf6: Y69R, V73K, and L77K; these amino acid changes impair the ability of E4orf6 to interact with Cul2 (20). We generated the DNA fragment for replacement by PCR amplification of FLAGmE4orf6TMWT8 with orfbTMFOR (5'ACTCACCATGTCAGCGACGC3') and orfbTMREV (5'TGGAATAAGGGGAGAAGAGCC3') and gel purification. We introduced this fragment into pKSB2.MAV-1colony16Δorf6TM::galK, selecting against GalK and selecting for the replacement with the E4orf6 mutant sequence by selecting for resistance to 2-deoxy-galactose (Recombineering Protocol 3; https://frederick.cancer.gov/resources/repositories/Brb/#/protocol and reference [71]). We prepared bacmid DNA using a purification method modified for BAC DNA (Origene NP10009). We identified a candidate colony C2 that had HindIII restriction pattern like the WTColony16 bacmid DNA and confirmed that it had the three E4orf6 aa changes by NruI digestion and by DNA sequencing a fragment PCR amplified using orfbTMFOR and orfbTMREV primers. We digested the MAV-1 genome from the bacmids using PmeI, gel purified, phenol-chloroform extracted, ethanol precipitated, and 2 μg was transfected into E4I5 cells using Lipofectamine 2000. After 5–8 days, suggestive signs of cytopathic effect were seen, and the cells plus media were harvested, freeze-thawed three times, and inoculated onto fresh E4I5 cells. After the third of such “blind” passages (each ~6–8 days), the lysate was titrated on 3T6 cells (described below). The titer was 1.1 × 10^4^ PFU/mL, and the stock was designated E4TMC2 (E4 triple mutant colony 2). After two additional passages on E4I5 cells, the E4TMC2 stock was passaged and titrated on E4I11 cells that had been induced with dexamethasone and used for subsequent experiments (titer 1.1 × 10^8^ PFU/mL). WT virus was titrated in parallel on E4I11 cells and had a titer of 1.5 × 10^8^ PFU/mL.

### Confirmation of E4TMC2 sequence

For isolation of viral DNA for sequence analysis from infected cells or viral stocks, we boiled 100 μL virus-containing cell culture supernatant and then incubated it with 10 μL Strataclean resin (Stratagene 400714/400715) for 1 min. The resin was removed by 15 second of centrifugation, and 1–2 μL of supernatant (containing viral DNA) was quantitated and used in 25–50 μL PCR reactions with primers from both strands flanking the mutations of E4orf6TMC2, orfbTMFOR and orfbTMREV. Sequencing with primers from both strands, orfbTMFOR and MAV27908 (5'TCATCCCCGGTCACGTAG3'), was performed by Eton Biosciences. The mutations of E4orf6TMC2 were confirmed by Sanger sequence analysis by comparing to WT virus sequence obtained in parallel.

To ensure that no off-target mutations occurred during the E4orf6TMC2 virus construction and isolation, whole-genome sequencing was performed by Azenta/GeneWiz. We prepared viral DNA using the Shinagawa method for WT and E4TMC2 virus (72). Bacmid DNAs corresponding to the WT and E4TMC2 were also sequenced. There were no sequence changes unique to the E4TMC2 viral or bacmid sequence other than the intended introduced mutations that change the predicted sequence of three amino acids in the Cul2-binding site region of the E4orf6 protein (20). Trimmed reads that mapped to the reference MAV-1 sequence (NC_000942) ranged in number from 749,000 to 4,100,000 with high-quality scores; thus, we are confident that there are no off-target mutations in the E4TMC2 virus. Upon obtaining the whole-genome sequence of the WT and mutant virus and bacmids, we noted 5 nt changes relative to the reference sequence NC_000942 that mapped to early region 2 (DNA polymerase and pTP) and late region 3 (hexon) in WT and mutant viral DNAs and their corresponding bacmids. We identified these changes in a genomic plasmid made from MAV-1 in 1985 in our lab. These changes were also found in MAV-1 sequence obtained and recently reported by another group (10). Thus, we conclude that NC_000942 has 5 nt sequencing errors.

### Quantitation of virus by plaque assay and determination of survival curves and LD_50_

We determined virus stock titers for WT virus on 3T6 cells as described (68). For the E4orf6TMC2 virus, we determined titers on E4I11 cells in media containing G418; for 24 h pre-infection and throughout the plaque assay, we included 10^−5^ M dexamethasone (detailed above). In experiments where WT and mutant viruses were compared, the titers used to determine MOI or mouse dose for both virus types were determined in parallel on the E4I11 complementing cell lines. This was because the titers of the WT virus on the complementing cell lines were approximately three- to sixfold higher than their titers on 3T6 cells, suggesting that dexamethasone treatment or the presence of E4 genes (E4orf6 or others) in the complementing cell lines stimulated WT viral growth.

For survival or LD_50_ analysis, groups of mice were infected with the indicated doses and monitored for up to 21 dpi. Mice that were moribund were euthanized by CO_2_ asphyxiation. LD_50_ values were calculated by the method of Reed and Muench (73).

### Quantitation of viral DNAs in mouse tissues

Brains and spleens were harvested, frozen, and DNAs were prepared using an Invitrogen PureLink Genomic DNA Kit (K182002). DNA (12.5 ng) was analyzed by qPCR with MAV-1 E1A genomic primers (74) in 15 μL reactions. A DNA standard curve was prepared from known amounts of an E1A gene-containing plasmid to convert cycle threshold values to E1A DNA copy numbers. Results were normalized to the amount of input DNA.

### Quantitation of mRNAs in mouse tissues

Tissue RNAs were prepared as described using Trizol and beadbeater disruption of tissue (24). cDNA was prepared using Applied Biosystems High-Capacity cDNA Reverse Transcription Kit (ThermoFisher 4368814). cDNAs were analyzed by SYBR green-based qPCR (Bio-Rad SYBR Green Supermix #1725121) in 10 μL reactions with 5 ng of DNA and intron-spanning primers for mouse actin (BetaActinUp and BetaActinDown [75]), MAV-1 E1A (59), MAV-1 late tripartite leader (76), or cellular β-IFN (77). Samples were analyzed on an Applied Biosystems 7500 Fast Real-Time PCR System, and reactions were 40 cycles of 15 s at 90°C and 60 s at 60°C. Target gene products were quantitated by normalizing to glyceraldehyde-3-phosphate dehdrogenase (GAPDH) and values expressed as 2^–ΔCt^, where Ct is the threshold cycle and ΔCt = Ct(target) – Ct(GAPDH).

### Antibodies

Two primary anti-PKR antibodies were used: mouse monoclonal anti-PKR B-10 (Santa Cruz Biotechnology, sc-6282) and rabbit monoclonal anti-PKR (Abcam, EPR19374). We used a mouse monoclonal to β-actin (Santa Cruz Biotechnology, sc-47778). Secondary antibodies for immunofluorescence were goat anti-rabbit Alexa Fluor 647 (Life Technologies, A21244) and, for immunoblot, goat anti-rabbit-HRP and goat anti-mouse HRP (Kindle Biosciences R1006, and 1005, respectively). Primary antibody to MAV-1 E4orf6 was 7871, a rabbit polyclonal antibody, described previously (7). Primary antibody to MAV-1 E1A was 10B10, a mouse monoclonal described previously (78).

### Immunofluorescence microscopy

Cells were grown on 12 mm glass coverslips (Thermo Fischer Scientific, 12–545-81) in 24-well plates. Cells were fixed in 4% (vol/vol) paraformaldehyde (Electron Microscopy Sciences, 15710) in DPBS for 15 min at room temperature, followed by permeabilization and blocking with 0.01% (wt/vol) saponin (Fluka BioChemika, 47036) and 3% bovine serum albumin (BSA; Sigma-Aldrich, A7906) in DPBS overnight at 4°C. The samples were incubated with primary antibody in 0.01% saponin and 3% BSA in DPBS overnight at 4°C, followed by incubation with secondary antibody and Hoechst 33342 solution diluted 1:10,000 (BD Biosciences, 561908) in saponin/BSA for 2 h at 4°C. Coverslips were mounted onto glass slides using ImmunoHistoMount mounting media (Sigma-Aldrich, I1161-30ML). Immunofluorescence was visualized using a Nikon Eclipse Ti2 widefield microscope (Michigan Medicine Microscopy Core) and Nikon NIS-Elements AR v. 5.42.03 acquisition software.

### Image analysis

Images were processed in FIJI v 1.54f (79) using equivalent settings. Histograms of four pictures, for both WT and PKR-TKO cell lines, were used to determine signal intensity ranges of 647 channel (PKR). From the compiled data, the lowest minimum and highest maximum values were identified, and the selected numbers were applied to rescale all original images. It may be possible to distinguish reflections or interference patterns resulting from UV light interacting with the coverslips for Hoechst images of WT cells in Fig. 1. This does not affect the interpretation of results.

### Preparation of cell lysates

For analysis of PKR in WT and PKR-TKO MEFs and BMDMs (Fig. 1), cells were plated and grown to 95% confluence on 12-well plates (MEFs, 3 × 10^5^ cells/well; and BMDMs, 1 × 10^6^ cells/well). At the time of harvest, cells were washed twice with cold DPBS, and then 500 μL 2× LGSB (BioRad 1610737) was added dropwise evenly across each well. The plates were rocked for 30 s, and then lysates were scraped and collected in 1.5 mL tubes. Samples were boiled 5 min before loading on gels. For analysis of viral yields in PKR-TKO MEFs (Fig. 2), we collected samples by cell scraping in their media at 24, 48, or 72 hpi. We harvested the produced virus particles by three freeze-thaw cycles of the cells, clarified by centrifugation at 889 × *g* for 10 min, and stored at −80°C until titration in 3T6 cells by plaque assay (68). For analysis of PKR in cells infected with the E4or6 mutant virus (Fig. 6), we plated 3T6 cells at 6 × 10^5^ cells per well in six-well plates and infected with WT or E4orf6 mutant virus at MOI of 5 or mock infected. At 24, 48, or 72 hpi, we washed cells twice in DPBS, scraped, pelleted them by centrifugation, and resuspended them in 100 μL of 2× LGSB. Samples were frozen and then boiled for 5 min prior to loading on gels.

### Immunoblot analysis

We prepared lysates for immunoblots as described above and analyzed them by SDS-PAGE on 4%–15% gradient gels (BioRad 4561085) that were then immunoblotted and visualized as described (7). Protein standards (Bio-Rad 1610374) were included in the gel as size markers.

### Sodium fluorescein assays

Male and female C57BL/6 or PKR-TKO mice, 3.5–4.5 weeks old, were infected for 8 days with 10^2^ PFU i.p. Ten minutes before euthanasia, mice were injected i.p. with 0.1 mL 10% (wt/vol) sodium fluorescein (Sigma F6377) in DPBS. Sera and brains were collected from the mice, and brains were snap-frozen on dry ice and then stored at −70°C until analysis. Sodium fluorescein in serum and right-brain hemispheres was determined as previously described (24). Fluorescence was measured with a Bio-Tek microplate reader (485 nm excitation, 530 nm emission) using sodium fluorescein standards prepared in DPBS. Brain fluorescence was normalized to the serum sample from each mouse, and the uptake ratio is (μg sodium fluorescein/g brain)/(μg sodium fluorescein/μL serum).

### Cytokine analysis

ELISA quantification of proteins was performed as described (24) and assayed by the University of Michigan Cancer Center Immunology Core. Approximately 80 mg of each brain was processed, or mouse serum was used directly. Samples were stored at −70°C until use when they were thawed on ice and centrifuged at 20,000 × *g* for 5 min at 4°C prior to assay.

### Histological analysis

After euthanasia, mice were perfused with 30 mL 10% neutral-buffered formalin, and organs were harvested and fixed in 10% neutral-buffered formalin for 48 h at 4°C and transferred to 70% ethanol for 24 h at 4°C. They were then paraffin-embedded, and 4 μm sections were stained with hematoxylin and eosin by the University of Michigan Tissue and Molecular Pathology Shared Resource. Slides were randomized and blinded for evaluation by a board-certified pathologist. The organ sections that were evaluated and histology scoring were done as described (24).

### Statistical analysis

GraphPad Prism 10.3.1 was used for statistical analyses.

## SUPPLEMENTAL MATERIAL

Fig. S1 to S3 jvi.01545-24-s0001.pdf Cytokine data and histopathology images.

ASM does not own the copyrights to Supplemental Material that may be linked to, or accessed through, an article. The authors have granted ASM a non-exclusive, world-wide license to publish the Supplemental Material files. Please contact the corresponding author directly for reuse.

## References

[1] Zhang R, Karijolich J. 2024. RNA recognition by PKR during DNA virus infection. J Med Virol 96:e29424. doi:10.1002/jmv.29424 38285432 PMC10832991

[2] Gal-Ben-Ari S, Barrera I, Ehrlich M, Rosenblum K. 2018. PKR: A kinase to remember. Front Mol Neurosci 11:480. doi:10.3389/fnmol.2018.00480 30686999 PMC6333748

[3] Luan X, Wang L, Song G, Zhou W. 2024. Innate immune responses to RNA: sensing and signaling. Front Immunol 15:1287940. doi:10.3389/fimmu.2024.1287940 38343534 PMC10854198

[4] Chaumont L, Collet B, Boudinot P. 2023. Double-stranded RNA-dependent protein kinase (PKR) in antiviral defence in fish and mammals. Dev Comp Immunol 145:104732. doi:10.1016/j.dci.2023.104732 37172664

[5] Kitajewski J, Schneider RJ, Safer B, Munemitsu SM, Samuel CE, Thimmappaya B, Shenk T. 1986. Adenovirus VAI RNA antagonizes the antiviral action of interferon by preventing activation of the interferon-induced eIF-2 alpha kinase. Cell 45:195–200. doi:10.1016/0092-8674(86)90383-1 3698097

[6] Goodman DE, Pretto CD, Krepostman TA, Carnahan KE, Spindler KR. 2019. Enhanced replication of mouse adenovirus type 1 following virus-induced degradation of protein kinase R (PKR). MBio 10:e00668-19. doi:10.1128/mBio.00668-19 31015330 PMC6479006

[7] Tejera-Hernández B, Goodman DE, Nevarez JM, Spindler KR. 2022. Mouse Adenovirus type 1 E4orf6 induces PKR degradation. J Virol 96:e0206321. doi:10.1128/jvi.02063-21 35285681 PMC9006929

[8] Spindler KR, Moore ML, Cauthen AN. 2007. Mouse adenovirusesp 49–65. In Fox JG, Barthold SW, Davisson MT, Newcomer CE, Quimby FW, Smith AL (ed), The mouse in biomedical research, 2nd ed. Vol. 2. Academic Press, New York.

[9] Hemmi S, Spindler KR. 2019. Murine adenoviruses: tools for studying adenovirus pathogenesis in a natural host. FEBS Lett 593:3649–3659. doi:10.1002/1873-3468.13699 31777948 PMC6928396

[10] Zhang Z, Guo X, Hou W, Zou X, Wang Y, Liu S, Lu Z. 2024. User-friendly replication-competent MAdV-1 vector system with a cloning capacity of 3.3 kilobases. Viruses 16:761. doi:10.3390/v16050761 38793642 PMC11126015

[11] Ball AO, Beard CW, Redick SD, Spindler KR. 1989. Genome organization of mouse adenovirus type 1 early region 1: a novel transcription map. Virology 170:523–536. doi:10.1016/0042-6822(89)90444-3 2543128

[12] Beard CW, Ball AO, Wooley EH, Spindler KR. 1990. Transcription mapping of mouse adenovirus type 1 early region 3. Virology 175:81–90. doi:10.1016/0042-6822(90)90188-w 2137954

[13] Kring SC, Ball AO, Spindler KR. 1992. Transcription mapping of mouse adenovirus type 1 early region 4. Virology 190:248–255. doi:10.1016/0042-6822(92)91211-c 1388309

[14] Lion T, Wold WSM. 2022. Adenoviruses. In Howley PM, Knipe DM (ed), Fields Virology, 7th ed. DNA Viruses. Lippincott Williams & Wilkins, Philadelphia.

[15] Procario MC, Levine RE, McCarthy MK, Kim E, Zhu L, Chang CH, Hershenson MB, Weinberg JB. 2012. Susceptibility to acute mouse adenovirus type 1 respiratory infection and establishment of protective immunity in neonatal mice. J Virol 86:4194–4203. doi:10.1128/JVI.06967-11 22345470 PMC3318603

[16] Weinberg JB, Stempfle GS, Wilkinson JE, Younger JG, Spindler KR. 2005. Acute respiratory infection with mouse adenovirus type 1. Virology 340:245–254. doi:10.1016/j.virol.2005.06.021 16054189 PMC1351061

[17] Hearing P. 2022. *Adenoviridae*: the viruses and their replication. In Howley PM, Knipe DM (ed), Fields virology, 7th ed. DNA Viruses. Lippincott Williams & Wilkins, Philadelphia.

[18] Price AM, Steinbock RT, Lauman R, Charman M, Hayer KE, Kumar N, Halko E, Lum KK, Wei M, Wilson AC, Garcia BA, Depledge DP, Weitzman MD. 2022. Novel viral splicing events and open reading frames revealed by long-read direct RNA sequencing of adenovirus transcripts. PLoS Pathog 18:e1010797. doi:10.1371/journal.ppat.1010797 36095031 PMC9499273

[19] Ball AO, Beard CW, Villegas P, Spindler KR. 1991. Early region 4 sequence and biological comparison of two isolates of mouse adenovirus type 1. Virology 180:257–265. doi:10.1016/0042-6822(91)90030-f 1845825

[20] Gilson T, Blanchette P, Ballmann MZ, Papp T, Pénzes JJ, Benkő M, Harrach B, Branton PE. 2016. Using the E4orf6-based E3 ubiquitin ligase as a tool to analyze the evolution of adenoviruses. J Virol 90:7350–7367. doi:10.1128/JVI.00420-16 27252531 PMC4984651

[21] Yang YL, Reis LF, Pavlovic J, Aguzzi A, Schäfer R, Kumar A, Williams BR, Aguet M, Weissmann C. 1995. Deficient signaling in mice devoid of double-stranded RNA-dependent protein kinase. EMBO J 14:6095–6106. doi:10.1002/j.1460-2075.1995.tb00300.x 8557029 PMC394734

[22] Abraham N, Stojdl DF, Duncan PI, Méthot N, Ishii T, Dubé M, Vanderhyden BC, Atkins HL, Gray DA, McBurney MW, Koromilas AE, Brown EG, Sonenberg N, Bell JC. 1999. Characterization of transgenic mice with targeted disruption of the catalytic domain of the double-stranded RNA-dependent protein kinase, PKR. J Biol Chem 274:5953–5962. doi:10.1074/jbc.274.9.5953 10026221

[23] Baltzis D, Li S, Koromilas AE. 2002. Functional characterization of PKR gene products expressed in cells from mice with a targeted deletion of the N terminus or C terminus domain of PKR. J Biol Chem 277:38364–38372. doi:10.1074/jbc.M203564200 12161430

[24] Castro-Jorge LA, Pretto CD, Smith AB, Foreman O, Carnahan KE, Spindler KR. 2017. A protective role for IL-1 signaling during mouse adenovirus type 1-induced encephalitis. J Virol 91:e02106-16. doi:10.1128/JVI.02106-16 27903802 PMC5286883

[25] Spindler KR, Fang L, Moore ML, Hirsch GN, Brown CC, Kajon A. 2001. SJL/J mice are highly susceptible to infection by mouse adenovirus type 1. J Virol 75:12039–12046. doi:10.1128/JVI.75.24.12039-12046.2001 11711594 PMC116099

[26] Welton AR, Chesler EJ, Sturkie C, Jackson AU, Hirsch GN, Spindler KR. 2005. Identification of quantitative trait loci for susceptibility to mouse adenovirus type 1. J Virol 79:11517–11522. doi:10.1128/JVI.79.17.11517-11522.2005 16103204 PMC1193630

[27] Guida JD, Fejer G, Pirofski L-A, Brosnan CF, Horwitz MS. 1995. Mus adenovirus type 1 causes a fatal hemorrhagic encephalomyelitis in adult C57BL/6 but not BALB/c mice. J Virol 69:7674–7681. doi:10.1128/JVI.69.12.7674-7681.1995 7494276 PMC189708

[28] Kajon AE, Brown CC, Spindler KR. 1998. Distribution of mouse adenovirus type 1 in intraperitoneally and intranasally infected adult outbred mice. J Virol 72:1219–1223. doi:10.1128/JVI.72.2.1219-1223.1998 9445021 PMC124599

[29] Kring SC, King CS, Spindler KR. 1995. Susceptibility and signs associated with mouse adenovirus type 1 infection of adult outbred Swiss mice. J Virol 69:8084–8088. doi:10.1128/JVI.69.12.8084-8088.1995 7494327 PMC189759

[30] Spindler KR, Welton AR, Lim ES, Duvvuru S, Althaus IW, Imperiale JE, Daoud AI, Chesler EJ. 2010. The major locus for mouse adenovirus susceptibility maps to genes of the hematopoietic cell surface-expressed LY6 family. J Immunol 184:3055–3062. doi:10.4049/jimmunol.0903363 20164425 PMC2832721

[31] Moore ML, McKissic EL, Brown CC, Wilkinson JE, Spindler KR. 2004. Fatal disseminated mouse adenovirus type 1 infection in mice lacking B cells or Bruton’s tyrosine kinase. J Virol 78:5584–5590. doi:10.1128/JVI.78.11.5584-5590.2004 15140955 PMC415807

[32] Gralinski LE, Ashley SL, Dixon SD, Spindler KR. 2009. Mouse adenovirus type 1-induced breakdown of the blood-brain barrier. J Virol 83:9398–9410. doi:10.1128/JVI.00954-09 19570856 PMC2738240

[33] Ashley SL, Pretto CD, Stier MT, Kadiyala P, Castro-Jorge L, Hsu TH, Doherty R, Carnahan KE, Castro MG, Lowenstein PR, Spindler KR. 2017. Matrix metalloproteinase activity in infections by an encephalitic virus, mouse adenovirus type 1. J Virol 91:e01412-16. doi:10.1128/JVI.01412-16 28053109 PMC5331797

[34] Beard CW, Spindler KR. 1996. Analysis of early region 3 mutants of mouse adenovirus type 1. J Virol 70:5867–5874. doi:10.1128/JVI.70.9.5867-5874.1996 8709206 PMC190604

[35] Hsu T-H, Althaus IW, Foreman O, Spindler KR. 2012. Contribution of a single host genetic locus to mouse adenovirus type 1 infection and encephalitis. MBio 3:e00131-12. doi:10.1128/mBio.00131-12 22647790 PMC3372963

[36] Moore ML, Brown CC, Spindler KR. 2003. T cells cause acute immunopathology and are required for long-term survival in mouse adenovirus type 1-induced encephalomyelitis. J Virol 77:10060–10070. doi:10.1128/jvi.77.18.10060-10070.2003 12941916 PMC224599

[37] Kaufman RJ. 1999. Double-stranded RNA-activated protein kinase mediates virus-induced apoptosis: a new role for an old actor. Proc Natl Acad Sci U S A 96:11693–11695. doi:10.1073/pnas.96.21.11693 10518510 PMC33789

[38] Lee SB, Esteban M. 1994. The interferon-induced double-stranded RNA-activated protein kinase induces apoptosis. Virology (Auckl) 199:491–496. doi:10.1006/viro.1994.1151 7510087

[39] Takizawa T, Ohashi K, Nakanishi Y. 1996. Possible involvement of double-stranded RNA-activated protein kinase in cell death by Influenza virus infection. J Virol 70:8128–8132. doi:10.1128/JVI.70.11.8128-8132.1996 8892939 PMC190888

[40] Yeung MC, Liu J, Lau AS. 1996. An essential role for the interferon-inducible, double-stranded RNA-activated protein kinase PKR in the tumor necrosis factor-induced apoptosis in U937 cells. Proc Natl Acad Sci U S A 93:12451–12455. doi:10.1073/pnas.93.22.12451 8901602 PMC38012

[41] García MA, Gil J, Ventoso I, Guerra S, Domingo E, Rivas C, Esteban M. 2006. Impact of protein kinase PKR in cell biology: from antiviral to antiproliferative action. Microbiol Mol Biol Rev 70:1032–1060. doi:10.1128/MMBR.00027-06 17158706 PMC1698511

[42] Price AM, Steinbock RT, Di C, Hayer KE, Li Y, Herrmann C, Parenti NA, Whelan JN, Weiss SR, Weitzman MD. 2022. Adenovirus prevents dsRNA formation by promoting efficient splicing of viral RNA. Nucleic Acids Res 50:1201–1220. doi:10.1093/nar/gkab896 34671803 PMC8860579

[43] Weber F, Wagner V, Rasmussen SB, Hartmann R, Paludan SR. 2006. Double-stranded RNA is produced by positive-strand RNA viruses and DNA viruses but not in detectable amounts by negative-strand RNA viruses. J Virol 80:5059–5064. doi:10.1128/JVI.80.10.5059-5064.2006 16641297 PMC1472073

[44] Richardson SJ, Willcox A, Hilton DA, Tauriainen S, Hyoty H, Bone AJ, Foulis AK, Morgan NG. 2010. Use of antisera directed against dsRNA to detect viral infections in formalin-fixed paraffin-embedded tissue. J Clin Virol 49:180–185. doi:10.1016/j.jcv.2010.07.015 20729142

[45] Cole JL. 2007. Activation of PKR: an open and shut case? Trends Biochem Sci 32:57–62. doi:10.1016/j.tibs.2006.12.003 17196820 PMC2703476

[46] Hesler S, Angeliadis M, Husain B, Cole JL. 2021. Contribution of dsRBD2 to PKR activation. ACS Omega 6:11367–11374. doi:10.1021/acsomega.1c00343 34056292 PMC8153938

[47] Lemaire PA, Lary J, Cole JL. 2005. Mechanism of PKR activation: dimerization and kinase activation in the absence of double-stranded RNA. J Mol Biol 345:81–90. doi:10.1016/j.jmb.2004.10.031 15567412

[48] Hovanessian AG, Galabru J. 1987. The double-stranded RNA-dependent protein kinase is also activated by heparin. Eur J Biochem 167:467–473. doi:10.1111/j.1432-1033.1987.tb13360.x 3653103

[49] Patel RC, Sen GC. 1998. PACT, a protein activator of the interferon-induced protein kinase, PKR. EMBO J 17:4379–4390. doi:10.1093/emboj/17.15.4379 9687506 PMC1170771

[50] Lee ES, Yoon CH, Kim YS, Bae YS. 2007. The double-strand RNA-dependent protein kinase PKR plays a significant role in a sustained ER stress-induced apoptosis. FEBS Lett 581:4325–4332. doi:10.1016/j.febslet.2007.08.001 17716668

[51] Carvalho-Filho MA, Carvalho BM, Oliveira AG, Guadagnini D, Ueno M, Dias MM, Tsukumo DM, Hirabara SM, Reis LF, Curi R, Carvalheira JBC, Saad MJA. 2012. Double-stranded RNA-activated protein kinase is a key modulator of insulin sensitivity in physiological conditions and in obesity in mice. Endocrinology 153:5261–5274. doi:10.1210/en.2012-1400 22948222

[52] Nakamura T, Furuhashi M, Li P, Cao H, Tuncman G, Sonenberg N, Gorgun CZ, Hotamisligil GS. 2010. Double-stranded RNA-dependent protein kinase links pathogen sensing with stress and metabolic homeostasis. Cell 140:338–348. doi:10.1016/j.cell.2010.01.001 20144759 PMC2820414

[53] Kitano T, Inagaki H, Hoshino S. 2024. The impact of single-stranded RNAs on the dimerization of double-stranded RNA-dependent protein kinase PKR. Biochem Biophys Res Commun 719:150103. doi:10.1016/j.bbrc.2024.150103 38761636

[54] Anderson BR, Muramatsu H, Nallagatla SR, Bevilacqua PC, Sansing LH, Weissman D, Karikó K. 2010. Incorporation of pseudouridine into mRNA enhances translation by diminishing PKR activation. Nucleic Acids Res 38:5884–5892. doi:10.1093/nar/gkq347 20457754 PMC2943593

[55] Smith K, Brown CC, Spindler KR. 1998. The role of mouse adenovirus type 1 early region 1A in acute and persistent infections in mice. J Virol 72:5699–5706. doi:10.1128/JVI.72.7.5699-5706.1998 9621028 PMC110240

[56] Brandt TA, Jacobs BL. 2001. Both carboxy- and amino-terminal domains of the vaccinia virus interferon resistance gene, E3L, are required for pathogenesis in a mouse model. J Virol 75:850–856. doi:10.1128/JVI.75.2.850-856.2001 11134298 PMC113981

[57] Blailock ZR, Rabin ER, Melnick JL. 1967. Adenovirus endocarditis in mice. Science 157:69–70. doi:10.1126/science.157.3784.69 4290792

[58] Blailock ZR, Rabin ER, Melnick JL. 1968. Adenovirus myocarditis in mice: an electron microscopic study. Exp Mol Pathol 9:84–96. doi:10.1016/0014-4800(68)90053-1 4299139

[59] McCarthy MK, Procario MC, Twisselmann N, Wilkinson JE, Archambeau AJ, Michele DE, Day SM, Weinberg JB. 2015. Proinflammatory effects of interferon gamma in mouse adenovirus 1 myocarditis. J Virol 89:468–479. doi:10.1128/JVI.02077-14 25320326 PMC4301126

[60] Spurgeon ME, Ornelles DA. 2009. The adenovirus E1B 55-kilodalton and E4 open reading frame 6 proteins limit phosphorylation of eIF2alpha during the late phase of infection.. J Virol 83:9970–9982. doi:10.1128/JVI.01113-09 19605483 PMC2747998

[61] Cesaro T, Michiels T. 2021. Inhibition of PKR by viruses. Front Microbiol 12:757238. doi:10.3389/fmicb.2021.757238 34759908 PMC8573351

[62] Herrmann C, Dybas JM, Liddle JC, Price AM, Hayer KE, Lauman R, Purman CE, Charman M, Kim ET, Garcia BA, Weitzman MD. 2020. Adenovirus-mediated ubiquitination alters protein-RNA binding and aids viral RNA processing. Nat Microbiol 5:1217–1231. doi:10.1038/s41564-020-0750-9 32661314 PMC7529849

[63] Balachandran S, Roberts PC, Brown LE, Truong H, Pattnaik AK, Archer DR, Barber GN. 2000. Essential role for the dsRNA-dependent protein kinase PKR in innate immunity to viral infection. Immunity 13:129–141. doi:10.1016/s1074-7613(00)00014-5 10933401

[64] Stewart MJ, Blum MA, Sherry B. 2003. PKR’s protective role in viral myocarditis. Virology (Auckl) 314:92–100. doi:10.1016/s0042-6822(03)00414-8 14517063

[65] Tushinski RJ, Oliver IT, Guilbert LJ, Tynan PW, Warner JR, Stanley ER. 1982. Survival of mononuclear phagocytes depends on a lineage-specific growth factor that the differentiated cells selectively destroy. Cell 28:71–81. doi:10.1016/0092-8674(82)90376-2 6978185

[66] Morgenstern JP, Land H. 1990. A series of mammalian expression vectors and characterisation of their expression of a reporter gene in stably and transiently transfected cells. Nucleic Acids Res 18:1068. doi:10.1093/nar/18.4.1068 2156225 PMC330385

[67] Chen C, Okayama H. 1987. High-efficiency transformation of mammalian cells by plasmid DNA modified title: high-efficiency transformation of mammalian cells by plasmid DNA. Mol Cell Biol 7:2745–2752. doi:10.1128/mcb.7.8.2745-2752.1987 3670292 PMC367891

[68] Cauthen AN, Welton AR, Spindler KR. 2007. Construction of mouse adenovirus type 1 mutants. Methods Mol Med 130:41–59. doi:10.1385/1-59745-166-5:41 17401163

[69] Tirumuru N, Pretto CD, Castro Jorge LA, Spindler KR. 2016. Mouse adenovirus type 1 early region 1A effects on the blood-brain barrier. mSphere 1:e00079-16. doi:10.1128/mSphere.00079-16 27303733 PMC4894691

[70] Warming S, Costantino N, Court DL, Jenkins NA, Copeland NG. 2005. Simple and highly efficient BAC recombineering using galK selection. Nucleic Acids Res 33:e36. doi:10.1093/nar/gni035 15731329 PMC549575

[71] Biswas K, Stauffer S, Sharan SK. 2012. Using recombineering to generate point mutations:galK-based positive-negative selection method. Methods Mol Biol 852:121–131. doi:10.1007/978-1-61779-564-0_10 22328430 PMC6668620

[72] Shinagawa M, Matsuda A, Ishiyama T, Goto H, Sato G. 1983. A rapid and simple method for preparation of adenovirus DNA from infected cells. Microbiol Immunol 27:817–822. doi:10.1111/j.1348-0421.1983.tb00638.x 6316118

[73] Reed LJ, Muench H. 1938. A simple method of estimating fifty per cent endpoints. Am J Hyg 27:493–497. doi:10.1093/oxfordjournals.aje.a118408

[74] Nguyen Y, McGuffie BA, Anderson VE, Weinberg JB. 2008. Gammaherpesvirus modulation of mouse adenovirus type 1 pathogenesis. Virology (Auckl) 380:182–190. doi:10.1016/j.virol.2008.07.031 PMC2577692 18768196

[75] Ashley SL, Welton AR, Harwood KM, Van Rooijen N, Spindler KR. 2009. Mouse adenovirus type 1 infection of macrophages. Virology (Auckl) 390:307–314. doi:10.1016/j.virol.2009.05.025 PMC2746394 19540545

[76] Molloy CT, Andonian JS, Seltzer HM, Procario MC, Watson ME, Weinberg JB. 2017. Contributions of CD8 T cells to the pathogenesis of mouse adenovirus type 1 respiratory infection. Virol (Auckl) 507:64–74. doi:10.1016/j.virol.2017.04.005 PMC5493992 28410483

[77] McCarthy MK, Procario MC, Wilke CA, Moore BB, Weinberg JB. 2015. Prostaglandin E2 production and T cell function in mouse adenovirus type 1 Infection following allogeneic bone marrow transplantation. PLoS ONE 10:e0139235. doi:10.1371/journal.pone.0139235 26407316 PMC4583312

[78] Fang L, Stevens JL, Berk AJ, Spindler KR. 2004. Requirement of Sur2 for efficient replication of mouse adenovirus type 1. J Virol 78:12888–12900. doi:10.1128/JVI.78.23.12888-12900.2004 15542641 PMC525005

[79] Schindelin J, Arganda-Carreras I, Frise E, Kaynig V, Longair M, Pietzsch T, Preibisch S, Rueden C, Saalfeld S, Schmid B, Tinevez J-Y, White DJ, Hartenstein V, Eliceiri KW, Tomancak P, Cardona A. 2012. Fiji: an open-source platform for biological-image analysis. Nat Methods 9:676–682. doi:10.1038/nmeth.2019 22743772 PMC3855844

