# Supplementary material for: Role of mouse adenovirus type 1 E4orf6-induced degradation of protein kinase R in pathogenesis
Source: J Virol. 2024 Dec 31;99(2):e01545-24. doi: 10.1128/jvi.01545-24 (PMC11852748; doi:10.1128/jvi.01545-24)

SUPPLEMENTAL FIGURE LEGENDS

Supplemental Fig. 1. Chemokine analysis of MAV-1-infected brains 8 dpi. The indicated cytokines were assayed by ELISA from technical duplicates of brain homogenates of MAV-1-infected or mock-infected WT and PKR-TKO mice. Data were analyzed by the Mann-Whitney test, comparing mock to infected (Inf) within each strain and by comparing MAV-1 infected strains. (*, $P < 0.05$; **, $P < 0.01$; ***, $P < 0.001$).

Supplemental Fig. 2. Chemokine analysis of E4orf6TMC2 mutant virus-infected brains. The same C57BL/6 mice in Fig. 7 that were infected i.p. with low or high doses of WT or E4orf6TMC2 (MUT) viruses were analyzed for chemokines by ELISA assay from technical duplicates of brain homogenates. The mean and standard deviation are shown. Significance was determined by one-way ANOVA, and the significant results of Tukey's multiple comparison tests are shown for comparisons by virus and by virus compared to mock infection. (*, $P < 0.05$; **, $P < 0.01$).

Supplemental Fig. 3. Histopathology of brains or hearts of mice infected with WT or mutant virus. B6 (WT) mice were mock infected or infected with WT or E4orf6TMC2 virus at the indicated doses and examined 8 dpi. Cortical brain (A-D) or heart (E-H) sections were prepared and stained by hematoxylin and eosin. (A, E) mock infected; (B, F) WT MAV-1, 10^2 PFU; (C, G) E4orf6TMC2 MAV-1, 10^7 PFU; (D, H) E4orf6TMC2 MAV-1, 10^2 PFU. A focal hemorrhage (magenta) is seen in C; lymphoplasmacytic myocarditis is seen in G. Size bars, 50 μM.

Supplemental Figure 1

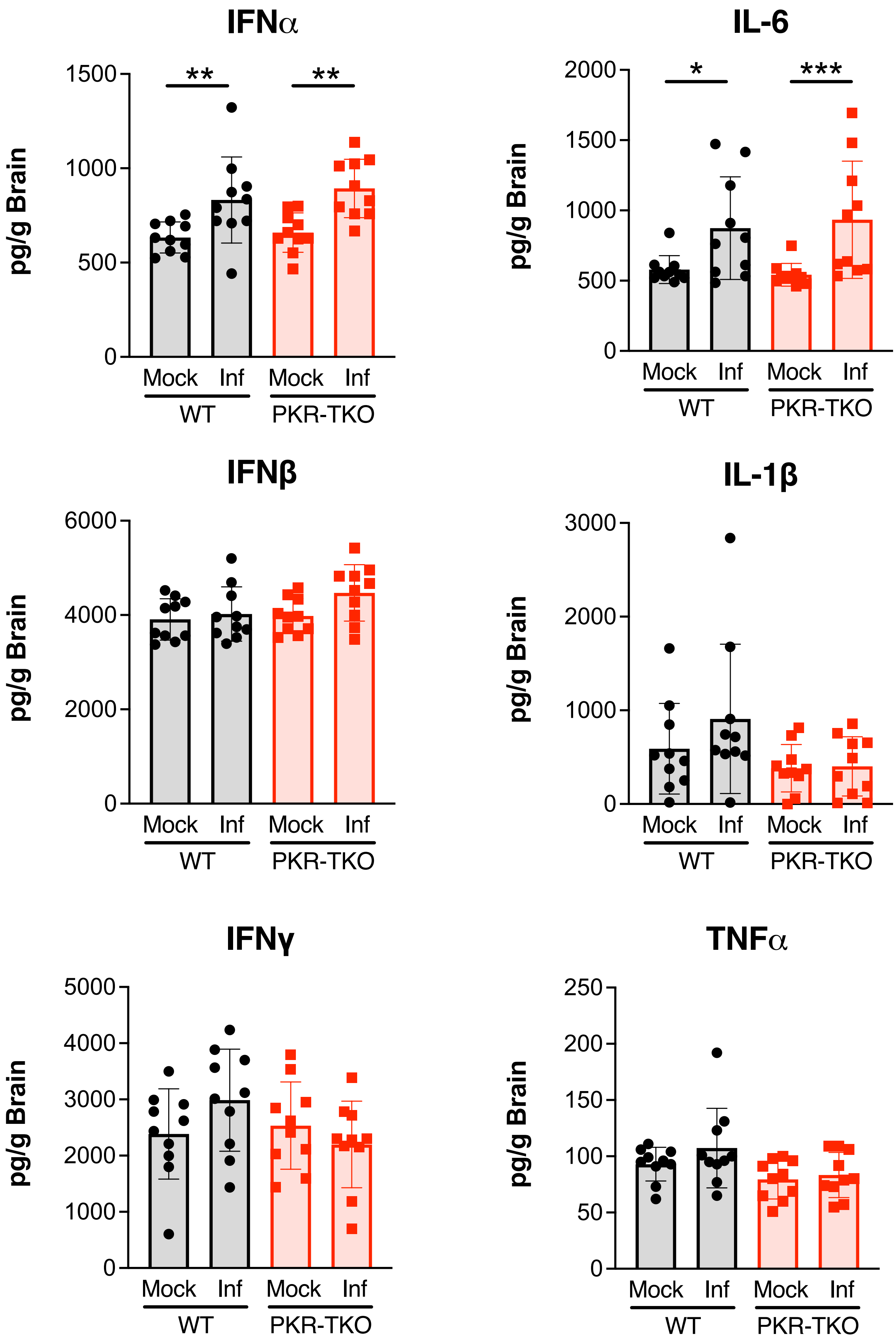

Supplemental Figure 2

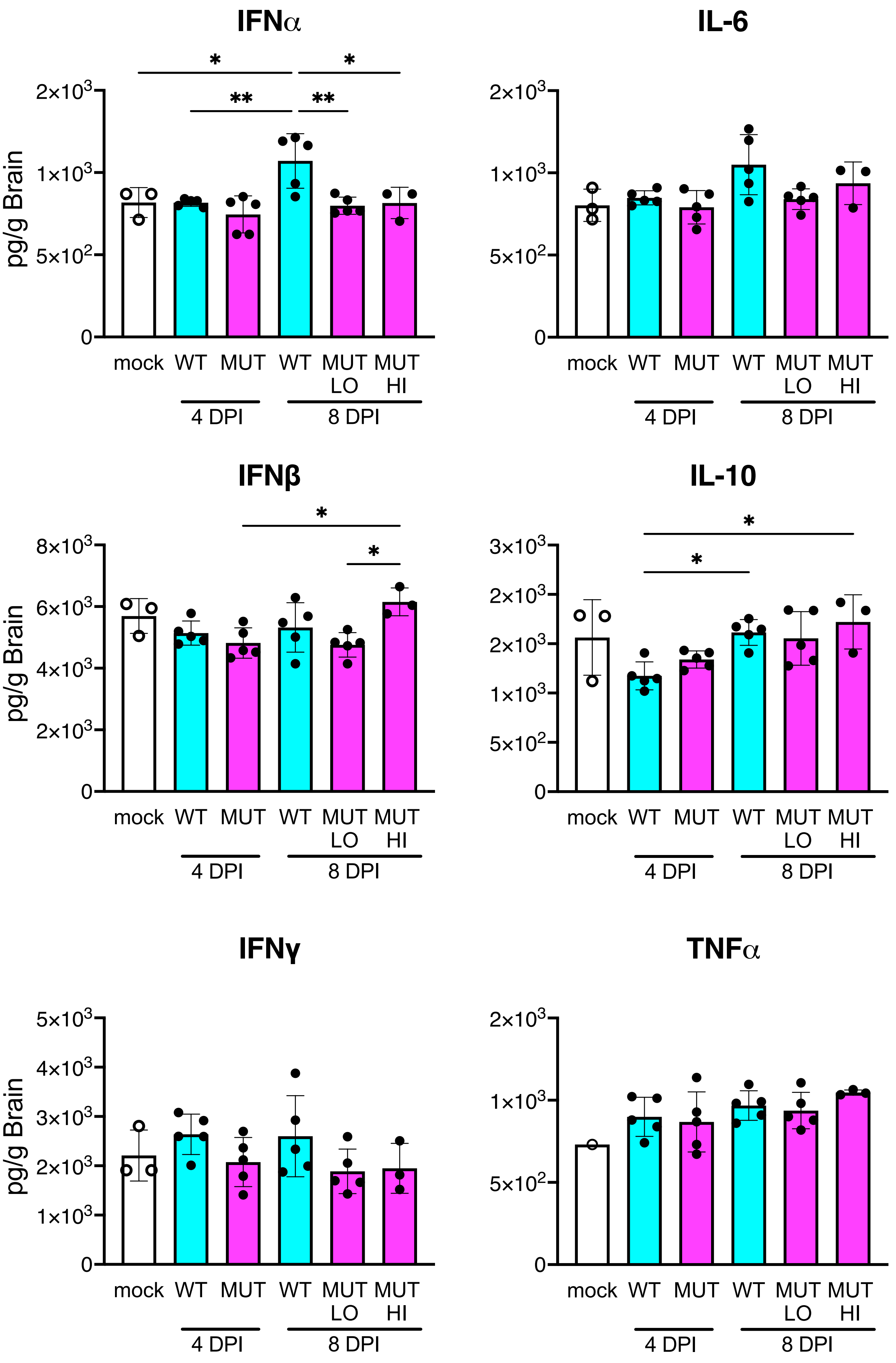

Supplemental Fig. 3

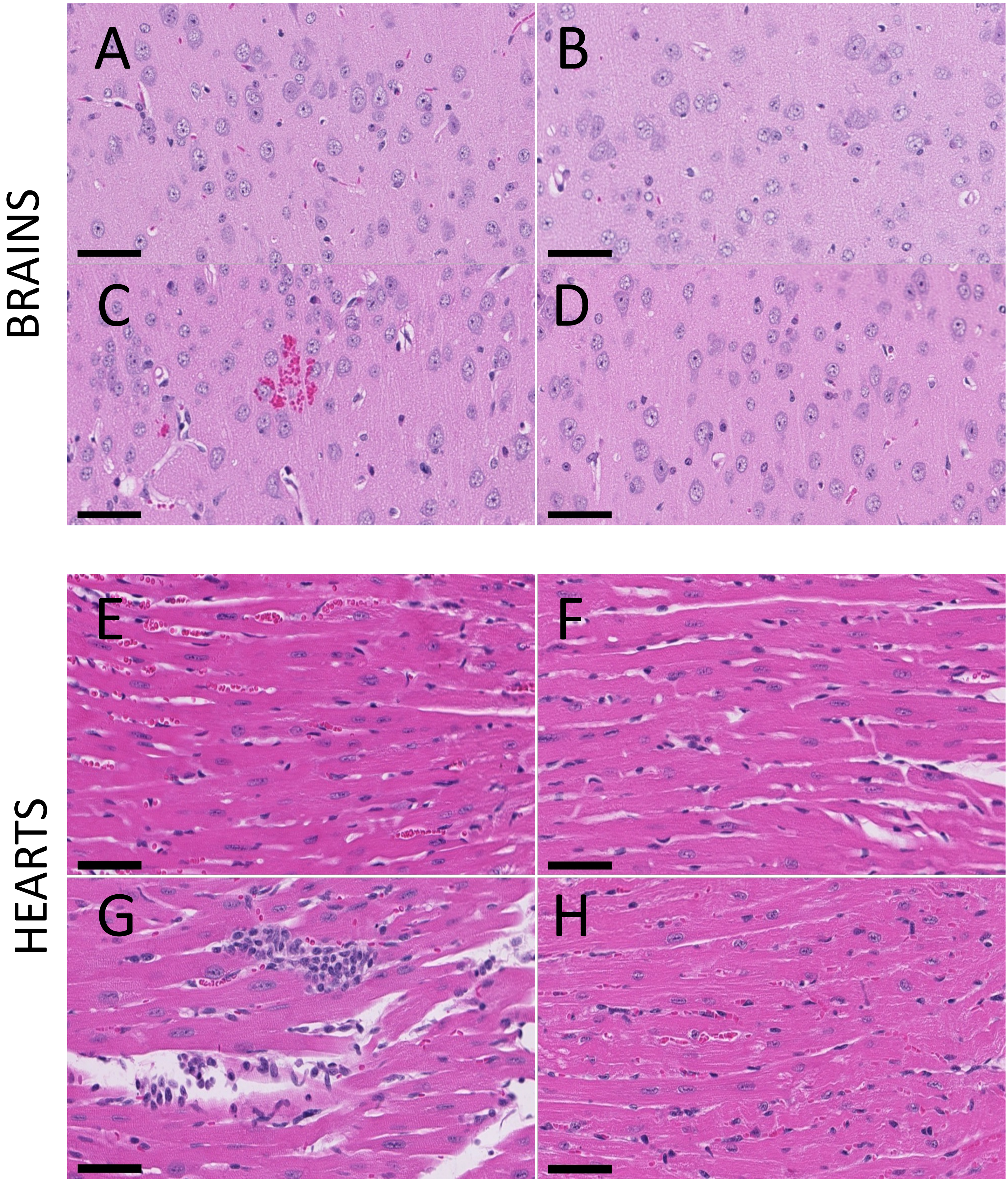

Supplement: Fig. S1 to S3 — Cytokine data and histopathology images. [file jvi.01545-24-s0001.pdf]
